# Total Release of 21 Indicator Pharmaceuticals Listed by the Swedish Medical Products Agency from Wastewater Treatment Plants to Surface Water Bodies in the 1.3 Million Populated County Skåne (Scania), Sweden

**DOI:** 10.3390/molecules27010077

**Published:** 2021-12-23

**Authors:** Erland Björklund, Ola Svahn

**Affiliations:** Department of Environmental Science and Bioscience, Kristianstad University, Elmetorpsvägen 15, SE-291 88 Kristianstad, Sweden

**Keywords:** indicator pharmaceuticals, wastewater, surface water

## Abstract

In 2017, the Swedish Environmental Protection Agency published a report on advanced wastewater treatment for the removal of pharmaceutical residues and stated that advanced treatment should be implemented where it will make the largest difference from an environmental perspective. However, the report also concluded that this need cannot be specified with existing data, but consideration must be made of local conditions. Two considerations are (1) the discharged amount of pharmaceutical into receiving water bodies and (2) the turnover of water in the recipient, where the highest risks are related to recipients with a low water turnover and low dilution. The current project comprised eight different WWTPs distributed throughout the entire County Skåne (Scania) in Sweden, with a population of ca. 1,300,000 persons. In total, 21 of 22 pharmaceuticals were analyzed according to the list proposed by the Swedish Medical Products Agency 2015. The results show that large amounts of pharmaceuticals are released from the WWTPs yearly to Scanian recipients. The total discharge of pharmaceuticals from the eight treatment plants adds up to 71 kg of these 21 substances alone, mainly comprising metoprolol, which is a drug that lowers blood pressure, and the analgesic drug diclofenac. Additionally, carbamazepine, losartan, naproxen and oxazepam were present in significant concentrations. These represented three illnesses that are very common: high blood pressure, inflammation/pain and depression/anxiety. The concentrations were generally in line with previous national Swedish screenings. It was estimated that, when one million cubic meters (1,000,000 m^3^) of wastewater is discharged, almost 4 kg of the 21 pharmaceuticals is released. The total volume wastewater release by the >90 WWTPs in Scania was estimated to 152,887,000 m^3^, which corresponded to 590 kg/year. The investigated 21 drugs cover only a small part of many hundred pharmaceuticals that are in use in Sweden. Thus, most likely, one or several tons of pharmaceuticals leak out to the Scanian recipients annually. The analysis of river samples shows that the dilution of wastewater is a key parameter in reducing concentrations. However, some locations have remarkably high concentrations, which occur when the volume wastewater is large in relation to the flow in the river. These kinds of regional results are of importance when selecting where advanced treatment should be prioritized in a first instance, as requested by the Swedish EPA.

## 1. Introduction

Pharmaceuticals in the environment (PIE) is an environmental topic that no longer can be considered new within the field of environmental contamination and toxicology. A well-known research paper with the title “Occurrence, fate and effects of pharmaceutical substances in the environment-A review” was published by Halling-Sørensen and coworkers more than 20 years ago and has today been cited over 4000 times [1]. Since then, a massive number of scientific articles have been released on basically all imaginable aspects of PIE [2,3,4,5,6,7,8,9,10,11,12,13,14]. Despite the large research funding spent on PIE, there are still many unknowns, as the topic is very complex. Nevertheless, today there is an increasing consensus among scientists that the present leakage of pharmaceuticals into the aquatic environment from our existing wastewater treatment plants (WWTPs) is unsustainable. It has been shown that some individual pharmaceuticals might be present in concentrations downstream WWTPs that could have adverse effects on wildlife, such as fish and birds [15,16,17]. Additionally, there is a concern that the overall effect of a large number of compounds released from WWTPs, in what often is referred to as a cocktail of micropollutants, may cause unwanted changes to receiving aquatic ecosystems [18,19]. Apart from these negative reports on the release of micropollutants from WWTPs, there is a parallel trend that this partly treated water could (and should) be seen as a future water resource, but more research and development is required to assure a safe use [20,21,22]. This is driven by a scarcity of water in many areas, as a consequence of increased water consumption by societies, combined with higher temperatures and draught due to a changing climate.

**Figure 1:**
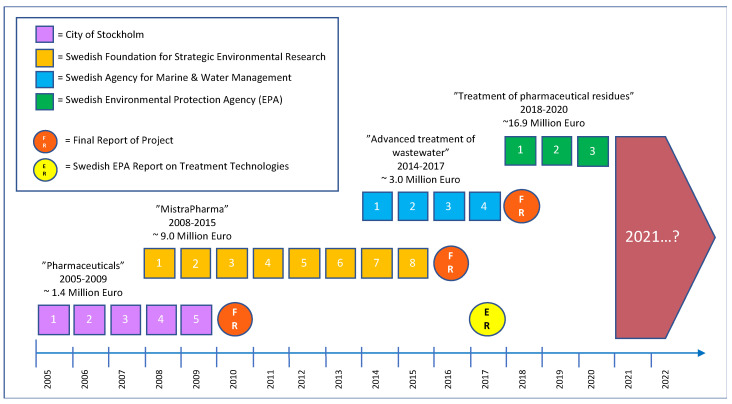
Example of major research funding made available in Sweden for 2005–2020 in relation to pharmaceuticals in the environment (PIE) and their removal from wastewater in WWTPs.

From a Swedish perspective, the awareness of the constant leakage of micropollutants, such as pharmaceuticals, to our recipients has been a topic of high public or political concern only in the past decade. Unlimited access to high-quality water has also always been nearly indisputable in Sweden, but after the summer of 2018, with extremely dry and hot weather conditions [23], the need for water increased. Especially agriculture was highlighted after historically low harvests. This justifies a better reuse of our limited water resources. In Sweden, several projects related to PIE have been performed in the past 15 years. Summarizing all of these is not the scope of this work; however, looking at some of the major funding made available during this period may be warranted, as it puts this and coming Swedish investigations into a broader context. It also points towards a change in how funding of PIE might (and possibly should) change from 2021 and onwards in order to make an actual impact by reducing the regional pollutant load to our recipients. Between 200 and 2009, a large Swedish project named “Pharmaceuticals-Presence and effects in the aquatic environment, preventive measures and possible treatment methods” was funded by Miljömiljarden, City of Stockholm, with a budget of 1.4 million Euro (Figure 1).

The final report was published in 2010 and concluded that there is a need to remove pharmaceuticals in Stockholm and that activated carbon or ozonation might be suitable technologies [24]. In 2008, a very general project called “MistraPharma” was launched, a project that, in the end, would receive a total of ~9.0 million Euros between 2008 and 2015 from the Swedish Foundation for Strategic Environmental Research (Figure 1). In its time, “MistraPharma” was one of largest research programs in the world within the PIE field. For 8 years, several research groups in Sweden tried to identify human pharmaceuticals that might be of concern to various aquatic ecosystems. “MistraPharma” also proposed risk management strategies, including better regulatory test requirements, as well as improved technologies for wastewater treatment. In the end, this resulted in 130 scientific papers, and, in 2016, the final report was published [25]. One of the main outcomes was 10 recommendations for improved environmental risk assessment [26]; however, some research was also dedicated to wastewater treatment technologies. Mainly two technologies showed to be promising: activated carbon and ozonation. As a logical continuation of these projects, the Swedish Agency for Marine and Water Management (on behalf of the Swedish Government) allocated ~3.0 million Euros to targeted projects that intended to develop novel techniques for “Advanced treatment of wastewater” from drug residues and other harmful compounds. The projects were operative between 2014 and 2017, mainly investigating ozonation and, to some extent, activated carbon (Figure 1). Most projects were headed by scientists, but the work was performed in collaboration with municipalities. Consequently, focus was partly moved from being primarily scientific to being more broadly connected to society by involving personnel at selected WWTPs. The final report was published in 2018 [27] and (once again) showed that ozonation and activated carbon were suitable to remove micropollutants and gave estimates of costs in doing so.

In parallel, the Swedish Government in December 2015 appointed the Swedish Environmental Protection Agency to find out if it would be conceivable to implement advanced treatment of wastewater to protect the aquatic environment from pharmaceuticals. This included analyzing which technical solutions were available and the pros and cons of these, as well as other effects that might occur if advanced treatment was introduced. The results were presented in a final report in 2017 [28] and concluded that the emission of pharmaceuticals can be reduced if Swedish WWTPs are equipped with more advanced technologies (Figure 1). However, it was notable that the Swedish EPA clearly stated that the Swedish society now must investigate where the technology should be introduced first, but concluded that, with existing occurrence data, this is not possible to specify. Several factors are important to make adequate prioritizations on where the needs are greatest, and consideration must be taken of local conditions, such as the following:

- The amount of pharmaceutical residues that are discharged into the recipients;
- The recipient’s water turnover;
- The number of WWTPs that discharge to the same recipient;
- The recipient’s sensitivity;
- Variations over the year;
- Variations in discharged amounts from the WWTP.

This is especially important as the Swedish Government, via the Swedish EPA, recently allocated a total of ~16.9 million Euros to fund different technological projects for “Treatment of pharmaceutical residues” at various WWTPs. This time, scientists at universities could be part of the projects but could not apply for the funding. Instead, the funding was now intended for Swedish municipalities who wanted to investigate advanced treatment technologies in a small or large scale on-site.

In order to harmonize the Swedish investigations, 22 pharmaceuticals were proposed as indicators by the Swedish Medical Products Agency in 2015 [29]. These pharmaceuticals should be analyzed and monitored in Swedish recipients. In our study, we utilized a method capable of analyzing all of these compounds simultaneously [30,31]. The year before, in 2014, the County Administrative Board of Scania released a document where guidelines concerning pharmaceuticals residues in wastewater were presented [32]. The document states that samples should be taken in outlet wastewater from WWTPs that are dimensioned for 200 PE or more, along with samples upstream and downstream the WWTP in question. Up to now, no Swedish or county studies have been performed at this large geographical resolution, as suggested by the County Administrative board of Scania [32], and, at the same time, employing the Swedish Medical Products Agency’s analytical protocol [29]. This paper attempts to start investigating where actions should be taken at a regional level as requested by the Swedish Environmental Protection Agency [28], as well as meeting the request from the Swedish Medical Products Agency [29] to estimate the burden of these indicator pharmaceuticals. This is important, since actual figures on burden will aid in the dialogue with politicians and stakeholder at local WWTPs as to why and where action should be taken to reduce the pharmaceutical release from Scanian WWTPs to the aquatic environment.

## 2. Results and Discussion

### 2.1. Wastewater Treatment Plants (WWTPs)

**Figure 2:**
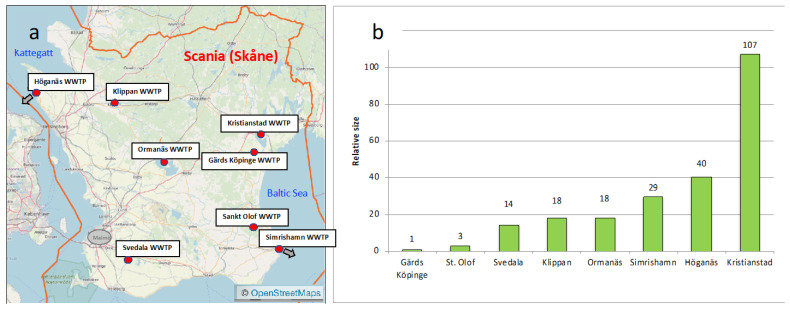
Eight investigated WWTPs. (**a**) Geographical spread of the WWTPs in Scania, Sweden. Gray arrows indicate that the WWTP discharges directly into the sea. (**b**) Relative size of the WWTPs based on annual volumes of treated wastewater attributing Gärds Köpinge WWTP a value of 1 (corresponding to approximately 77,000 m^3^ treated water/year).

The eight WWTPs were distributed over the entire County Scania (Figure 2a), and the annual volumes of treated wastewater differed largely, as seen in Supplementary Materials, Table S1a. The extent of variation ranged from 77,000 m^3^ in the tiniest WWTP in Gärds Köpinge to 8,000,000 m^3^ at Kristianstad WWTP. The relative size of the eight WWTPs, using the annual volume of treated wastewater as a base and thereafter attributing Gärds Köpinge WWTP a value of 1, showed that the WWTPs varied by a factor more than 100, as seen in Figure 2b.

The water flow expressed as an average in m^3^/h varied by a factor of 109 from Gärds Köpinge WWTP, at 8.75 m^3^/h, to Kristianstad WWTP, at 958 m^3^/h. The PE differed from 425 to 118,300 PE, corresponding to a factor of 278. Kristianstad WWTP and Simrishamn WWTP stood out, as they contain a large proportion industrial water. Consequently, the included WWTPs represented very different types of wastewater treatment plants. Complementary information about the treatment techniques applied in the different WWTPs can be found in Supplementary Materials, Table S1b, which is based on the information provided by the participants in the questionnaires.

### 2.2. Results of Pharmaceutical Analyses

#### 2.2.1. Chemical Emissions of Pharmaceuticals as Concentrations (ng/L)

**Figure 3:**
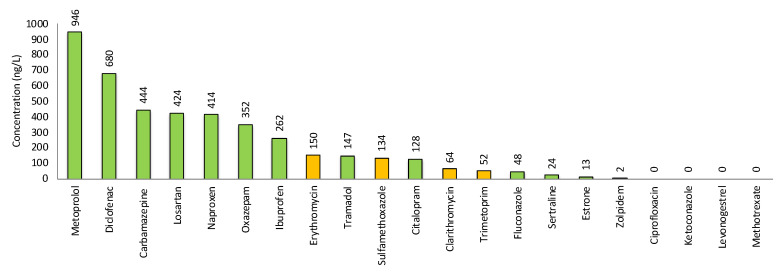
Average concentrations (ng/L) of the 21 analyzed pharmaceuticals in outlet water from 8 Scanian WWTPs (see Supplementary Materials Table S2). Antibiotics are marked in orange, except for ciprofloxacin, which was not found in measurable concentrations.

The recipients’ chemical loads, expressed as outlet concentrations of pharmaceuticals, from all eight WWTPs are presented in Supplementary Materials, Table S2. From these data, the average emission concentration from the eight Scanian WWTPs of each specific pharmaceutical was calculated in order to get an overview of which substances that had the highest concentrations in Scanian wastewater in general, as seen in Figure 3.

Based on this information, the compounds are discussed below in groups based in outlet concentrations.

##### Metoprolol, Diclofenac, Carbamazepine, Losartan, Naproxen, Oxazepam and Ibuprofen

These compounds comprise several types of pharmaceuticals, representing three general and common illnesses: (1) high blood pressure, (2) inflammation/pain and (3) depression/anxiety. The two top pharmaceuticals were metoprolol and diclofenac, occurring at average concentrations between 500 and 1000 ng/L, followed by five pharmaceuticals (carbamazepine, losartan, naproxen, oxazepam and ibuprofen) occurring between 250 and 499 ng/L.

**Figure 4:**
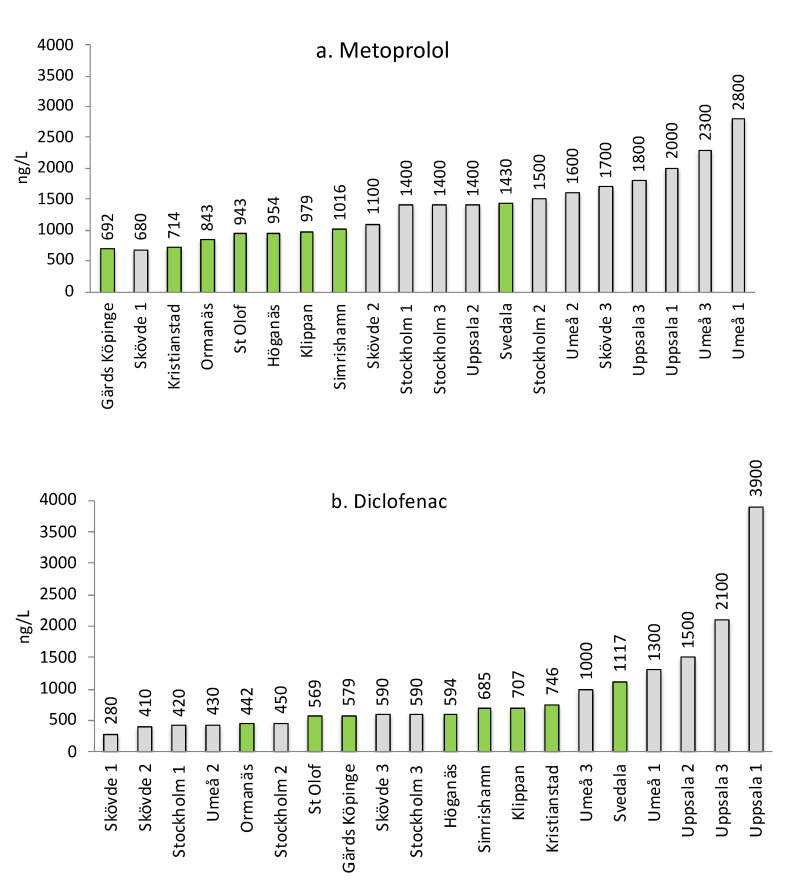
Measured concentrations (ng/L) of (**a**) metoprolol and (**b**) diclofenac in outlet water from the 8 Scanian WWTPs and in the Swedish National Screening Programme (Report 2011) for the cities Skövde, Stockholm, Uppsala and Umeå. The WWTPs in these 4 cities were analyzed three times each, as indicated by 1, 2 and 3. The 8 Scanian WWTPs are shown in green bars.

Metoprolol is a ß-blocker used against high blood pressure and is well-known to be recalcitrant during conventional wastewater treatment [33]. Metoprolol had the highest average concentration of all substances at 946 ng/L (1 µg/L). The variation among the WWTPs was small. The lowest concentration was observed in Gärds Köpinge, at 692 ng/L, while Svedala had the highest value, at 1430 ng/L. The concentrations are close to those measured in earlier Swedish studies. In 2010, Stockholm Vatten showed that Henriksdal WWTP had an outlet concentration of 1161 ng/L (RSD = 53%), whereas Bromma WWTP had 1320 ng/L (RSD = 68%) [24]. Several WWTPs were also tested in a Swedish National Screening Programme in 2011 (National Screening), where outlet concentrations from WWTPs at the four cities, i.e., Skövde, Stockholm, Uppsala and Umeå, were reported [34]. A comparison of metoprolol between the eight Scanian WWTPs and theses four cities is shown in Figure 4a.

The concentrations in the Scanian WWTPs are on a par or somewhat below the results from the National Screening. It should be noted that no Scanian WWTPs were included in either Stockholm Vatten [24] or in National Screening [34], thus highlighting the need for a regional assessment of the pharmaceutical load in this highly populated Swedish area. Metoprolol was also recently identified at up to 10 ng/L in the large Swedish lake Mälaren, showing that this compound is present in the Swedish aquatic environment [35].

Diclofenac is a NSAID belonging to a group of anti-inflammatory drugs and has shown to be semi-persistent in the Swedish environment [36]. Swedish researchers recently demonstrated that, when three-spine stickleback (*Gasterosteus aculeatus*) was exposed to even low μg/L concentrations of diclofenac, it caused histological changes, and the researchers expressed concern about fish populations exposed to treated sewage effluents [37]. Diclofenac was recently also highlighted by the Swedish Medical Products Agency for being so heavily used and easily accessible in Sweden [38]. The total Swedish sales amounted to 4.5 tons, with 2.6 tons OTC and 1.9 tons on prescription. In Scania, more than 600 kg were sold, with 50% OTC. In our study, diclofenac presented the second highest concentration with an average concentration of 680 ng/L (0.7 µg/L) and a small variation between WWTPs. The lowest concentration was observed at Ormanäs, at 442 ng/L, while the highest concentration was observed at Svedala, at 1117 ng/L. Stockholm Vatten reported that the outlet concentration in the Henriksdal WWTP was 288 ng/L (RSD = 36%), while, in the Bromma WWTP, it was 257 ng/L (RSD = 32%) [24]. When comparing with the National Screening [34] (Figure 4b) pronounced similarities in concentrations are observed. In an EU-wide monitoring covering 90 WWTPs (EU Monitoring) [39], the detection frequency was 89%, the maximum concentration 174 ng/L and the average concentration 49 ng/L. These are somewhat lower concentrations; however, the authors commented that their diclofenac concentrations might be underestimated. The concentrations of metoprolol and diclofenac in the eight Scanian WWTPs are compared in Supplementary Materials, Figure S1: Figure S1a shows the similarities in measured concentrations in outlet wastewater, while Figure S1b shows that the size of the WWTP has no influence on the outlet concentrations. Diclofenac was also recently quantified in Swedish lake Mälaren at 3.5 ng/L [35].

Carbamazepine is administered to treat epilepsy and alcohol abstinence and has a well-known persistence in water [40]. Carbamazepine occurred in all WWTPs between 139 ng/L (Gärds Köpinge) and 699 ng/L (Svedala), with an average concentration of 444 ng/L. Stockholm Vatten reported a concentration of 373 ng/L (RSD = 32%) at Henriksdal and 305 ng/L at Bromma (RSD = 35%) [24]. The concentrations in the four cities in National Screening [34] varied between 460 and 1100 ng/L, while the EU Monitoring had a detection frequency of 90%, a maximum concentration 4609 ng/L and a somewhat higher average concentration of 832 ng/L [39]. Concentrations in Scania are in line with previous Swedish screenings, but somewhat lower than the average concentration in Europe. In a recent Swedish study covering the entire Baltic Sea, carbamazepine was identified with a frequency of >90% in 43 samples, as a consequence of extensive usage, low removal in WWTPs and environmental persistence [41]. The low detection limits, in combination with its persistence, have made carbamazepine a good indicator of wastewater intrusion in natural waters [42]. The environmental concentrations in Swedish lake Mälaren reached as high as 20 ng/L [35].

Losartan is a blood-pressure-lowering medicine. Recently, researchers observed effects on the brown mussel (*Perna perna*), even at ng/L levels, and suggested it as a suitable marine model in environmental assessments [43]. The average concentration in Scanian WWTPs was 424 ng/L, with a relatively high variation; Ormanäs had the lowest concentration, at 83 ng/L; and Svedala had the highest, at 921 ng/L. The concentration at Henriksdal was 204 ng/L (RSD = 48%), and at Bromma, it was 187 ng/L (RSD = 48%), according to Stockholm Vatten [24]. Losartan was neither analyzed in the National Screening [34] nor in EU Monitoring [39], but the concentrations in Scania are within a factor of two compared to Stockholm Vatten [24]. Losartan was quantified at 5 ng/L in lake Mälaren revealing its environmental occurrence [35].

Naproxen is an anti-inflammatory NSAID pharmaceutical. Swedish researchers recently pointed out that naproxen and diclofenac produce highly similar toxic effects in fish, but based on new experiments, they showed that the environmental hazards and risks are lower for naproxen [44]. Hence, they stated that substitution would be advisable when naproxen presents an adequate alternative from a clinical point-of-view. The Scanian WWTPs had an average concentration of 414 ng/L. The concentrations vary in a way that resembles losartan with the lowest concentration of 119 ng/L at Kristianstad and the highest of 1430 ng/L at Sankt Olof. The concentration in Sankt Olof is much higher than in the other WWTPs, considering that the Simrishamn has the second highest value of only 379 ng/L. Excluding Sankt Olof reduces the average concentration to 269 ng/L. Sankt Olof is one of the smallest WWTPs, and an explanation for the high value could be a high consumption of naproxen in this small population during this explicit sampling event, causing extraordinary concentrations this day. The concentration at Henriksdal was 476 ng/L (RSD = 84%), and at Bromma, it was 565 ng/L (RSD = 46%), as shown by Stockholm Vatten [24]. The concentrations of naproxen in the four cities investigated in National Screening [34] ranged between 26 and 490 ng/L. The EU Monitoring reported a detection frequency of 66%, while maximum and average concentration were 958 and 27 ng/L, respectively [39]. Just as for diclofenac, this might be an underestimate. In general, the concentrations in Scania were close to previous Swedish studies.

Oxazepam is a benzodiazepine administered to treat anxiety and depression. It is classified as a narcotic and is well-known for its observed effects on perch (*Perca fluviatilis*) [16] and common roach (*Rutilus rutilus*) [45], as Swedish researchers demonstrated behavior changes in these fish species when exposed to low µg/L levels. The average concentration was 352 ng/L, with a low in Gärds Köpinge of 95 ng/L and a high in Kristianstad of 475 ng/L. Gärds Köpinge stands out with a 3–4 times lower concentration. Stockholm Vatten reported concentrations at Henriksdal of 324 ng/L (RSD = 49%), and at Bromma of 190 ng/L (RSD = 31%) [24]. A comparison between the four cities in National Screening [34] and the eight Scanian WWTPs is shown in Supplementary Materials, Figure S2. The average concentration of these four cities was 463 ng/L, which agrees well with Scania. The detection frequency in the EU Monitoring was 90% and a maximum concentration of 1766 ng/L [39]. The average concentration was lower (162 ng/L), but within a factor of two compared to Scania. Thirteen benzodiazepines were monitored in a study including 30 European rivers [46]. In the study, oxazepam had the highest detection frequency (85%), with a maximum concentration of 61 ng/L, showing widespread environmental occurrence. The Swedish lake Mälaren also contained oxazepam up to 5 ng/L [35].

Ibuprofen is a third NSAID drug (similar to diclofenac and naproxen) and is available as an OTC pharmaceutical with a widespread use. The average concentration was 262 ng/L; however, the differences in observed concentrations were very large. At Gärds Köpinge, Höganäs and Kristianstad WWTPs ibuprofen could not be detected. In contrast, at Ormanäs the observed concentration exceeded 1 µg/L (1158 ng/L). Stockholm Vatten found a concentration of 42 ng/L (RSD = 136%) at Henriksdal and 80 ng/L (RSD = 108%) at Bromma [24]. The ibuprofen concentrations varied largely in National Screening [34] just as they did in the Scanian WWTPs, with concentrations from 42 to 990 ng/L in the four cities. The EU Monitoring showed a detection frequency of 57%, with maximum and average concentrations of 2129 and 81 ng/L, respectively [39]. This is comparable to Scanian observations, where both frequency and concentration vary largely between WWTPs.

##### Seasonal Variation in Concentrations of the Top Six Pharmaceuticals at Kristianstad WWTP

**Figure 5:**
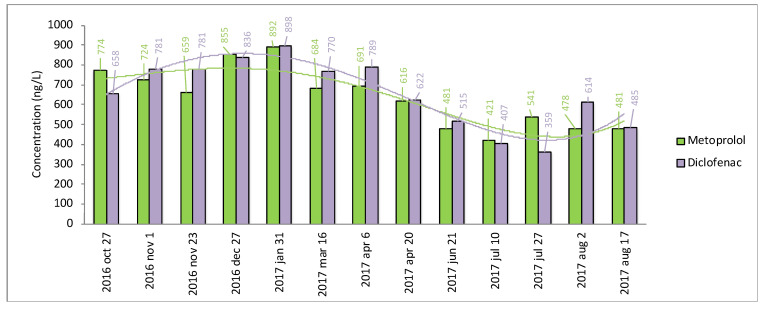
Measured concentrations (ng/L) of metoprolol and diclofenac in outlet water from Kristianstad WWTP during the season 2016/2017 (*n* = 13).

To investigate how pharmaceutical concentrations vary over time and disclose the representativeness of the selected sampling period (April), a study was conducted at Kristianstad WWTP. Samples were taken at 13 occasion to cover all four seasons. The top six pharmaceuticals, which all occurred in all WWTPs (Supplementary Materials, Table S2), were analyzed in these samples. The results are seen in Supplementary Materials, Table S3, while the seasonal variation is shown in Figure 5 for metoprolol and diclofenac. This shows a tendency towards highest concentrations in December/January and a slight drop in June/July to then increase again. Similar results were observed for all six pharmaceuticals (Supplementary Materials, Figure S3). Notably, metoprolol had an average annual concentration of 638 ng/L (*n* = 13; Supplementary Materials, Table S3), which is close to the concentration of metoprolol at Kristianstad WWTP, with a value of 714 ng/L (*n* = 1; see Supplementary Materials, Table S2). The month of April thereby seems to mirror the annual average concentration for Kristianstad WWTP reasonably well. The similarities seen for metoprolol between the month of April (*n* = 1) and the entire season 2016/2017 (*n* = 13), were also observed for diclofenac (746 vs. 655 ng/L), carbamazepine (470 vs. 441 ng/L), losartan (217 vs. 219 ng/L), naproxen (119 vs. 173 ng/L) and oxazepam (475 vs. 499 ng/L).

##### Antibiotics

Five antibiotics were analyzed: ciprofloxacin, clarithromycin, erythromycin, sulfamethoxazole and trimethoprim (Supplementary Materials, Table S2 and Figure 3).

Ciprofloxacin is an antibiotic (broad-spectrum) used to treat bacterial infections of various types. Ciprofloxacin is known to adsorb to the sludge phase [47], and this probably explains why it was not detected in any wastewater outlets. In previous Swedish studies, ciprofloxacin has been quantified in sludge up to a few thousand µg/kg [47,48]. Stockholm Vatten [24] reported low concentrations of 20 ng/L (RSD = 50%) at Henriksdal and 40 ng/L (RSD = 44%) at Bromma. The concentrations in the for the four cities in National Screening [34] ranged from 0 up to 65 ng/L. In the EU Monitoring [39], the detection frequency was 90% and the maximum concentration found was 264 ng/L, while the average concentration was 96 ng/L. In the most recent Swedish study [47] covering 11 WWTPs, the detection frequency in treated effluent water was 27% with maximum and average concentrations of 62 and 38 ng/L, respectively. This is similar to a previous Swedish study including five WWTPs, where the outlet concentration varied between <LOQ and 60 ng/L [49]. Overall, the reported ciprofloxacin outlet concentrations in Swedish WWTPs are below 70 ng/L.

Clarithromycin and erythromycin are both macrolides with a chemical structure that is very similar. Both are administered to treat bacterial infections. Clarithromycin concentrations ranged from <LOQ to 213 ng/L and erythromycin from 1 to 640 ng/L. Stockholm Vatten [24] did not analyze clarithromycin, while the concentration of erythromycin was 236 ng/L (RSD = 67%) which can be compared to the Scanian average concentration of 151 ng/L. Noteworthy is that in the two smallest WWTPs Gärds Köpinge and Sankt Olof, erythromycin was only found in low concentrations of <3 ng/L which is discussed in detail below. By removing Gärds Köpinge and Sankt Olof from the average calculation, a new average of 200 ng/L is obtained for Scania, which is close to Stockholm Vatten [24]. In National Screening [34], clarithromycin varied in the range of <LOQ–780 ng/L, and erythromycin varied between 53 and 530 ng/L. Both were close to the Scanian WWTPs, but the range was broader. In the Swedish study including 11 WWTPs, the detection frequencies were 61% and 88% for clarithromycin and erythromycin, respectively [47]. Clarithromycin had maximum and average concentrations of 86 and 36 ng/L, respectively, while corresponding concentrations for erythromycin were 350 and 129 ng/L, which is in line with this study. None of the macrolides was analyzed in the EU Monitoring [39].

Sulfamethoxazole and Trimethoprim are used in combination. The ratio of SMX/TMP in raw wastewaters has been suggested as a marker of wastewater origin where hospital effluents and WWTP influents show similar SMX/TMP footprints, while livestock effluents exhibit higher SMX/TMP ratios, due to usage of SMX alone in farming [50]. Recently researchers investigated the ecotoxic effects of sulfamethoxazole–diclofenac in a binary mixture to bacteria (*A. fischeri*), crustaceans (*D. magna*) and vascular plants (*L. minor*) [51]. Sulfamethoxazole alone showed low toxicity with low environmental risk, while diclofenac revealed moderate toxicity with a high risk to aquatic organisms. However, the mixture demonstrated the highest toxicity to the exposed model organisms. Nevertheless, when studying the toxicity of synthetic wastewater, no increase in toxicity was seen for wastewater spiked with sulfamethoxazole and diclofenac. Consequently, complex interactions likely occur between matrix components in environmental samples, thus affecting the wastewater toxicities that are observed. The concentrations in the eight Scanian WWTPs varied from a few to 281 ng/L, with average concentrations of 134 ng/L for sulfamethoxazole and 52 ng/L for trimethoprim. Both compounds were detected at Henriksdal at 60 ng/L (RSD = 67%) and 35 ng/L (RSD = 45%), and at Bromma with concentrations of 52 ng/L (RSD = 54%) and 186 ng/L (RSD = 29%), respectively, by Stockholm Vatten [24]. National Screening [34] quantified sulfamethoxazole between 30 and 290 ng/L and trimethoprim between 60 and 510 ng/L. Pharmaceutical emissions from Scanian WWTPs are similar to Stockholm Vatten [24] but lower than National Screening [34]. The outlet concentration of sulfamethoxazole and trimethoprim in five Swedish WWTPs ranged between <LOQ and 304 ng/L and between 66 and 1340 ng/L, respectively [49]. In the EU Monitoring, two different results were reported for sulfamethoxazole [39]: (I) detection frequency of 83% with maximum and average concentrations of 1691 and 280 ng/L, respectively; and (II) detection frequency of 81% with maximum and average concentrations of 1147 and 142 ng/L, respectively. For trimethoprim the detection frequency was 93%, with a maximum concentration of 800 ng/L and an average concentration of 229 ng/L. In general, these concentrations were higher than those reported in Scania.

**Figure 6:**
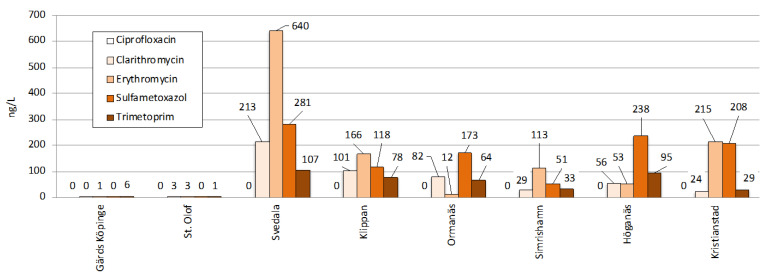
Measured outlet water concentrations (ng/L) of the five antibiotics, namely ciprofloxacin, clarithromycin, erythromycin, sulfamethoxazole and trimethoprim, in the 8 Scanian WWTPs.

Antibiotics and size of WWTP: The analyzed antibiotics did not show any obvious relation between concentrations and size of WWTP. However, a concrete difference was observed between the two WWTPs in Gärds Köpinge and Sankt Olof (the smallest WWTPs) and the six other WWTPs (Figure 6).

These two WWTPs are a factor 5–30 smaller compared to the three in order (Figure 2b), and the number of PE is only 425 in Gärds Köpinge and 600 in Sankt Olof. This can possibly be explained by antibiotics not being consumed as regularly as, for example, heart medication. Instead, these antibiotics are taken as specific courses of treatment for a limited time. Small WWTPs seldom have enough parallel antibiotic treatments to allow detection.

##### Tramadol, Citalopram, Fluconazole and Sertraline

The four pharmaceuticals, i.e., tramadol, citalopram, fluconazole and sertraline, had average concentrations in the range of 24–147 ng/L for the eight Scanian WWTPs (Figure 3 and Supplementary Materials, Table S2).

Tramadol is an opioid and classed as a narcotic in Sweden. Tramadol has previously been investigated in 33 Swedish WWTPs’ influent wastewater, together with 23 other illicit drugs [52]. Tramadol was detected in 100% of the samples, showing prevalent usage. In Scanian WWTPs, the concentrations were between 81 and 208 ng/L, with an average concentration of 147 ng/L. This differs from Henriksdal and Bromma, with concentrations of 571 ng/L (RSD = 49%) and 474 ng/L (RSD = 50%), respectively, Stockholm Vatten [24]. Even higher concentrations were observed in National Screening between 730 and 3000 ng/L [34]. It is noteworthy that tramadol showed one of the largest measurement uncertainties among five laboratories in a recent inter-calibration exercise that was headed by us on assignment of the Swedish Agency for Marine and Water Management [53]. This might explain part of this large variation. The EU Monitoring [39] reported a detection frequency of 100% with maximum and average concentrations of 1166 and 256 ng/L, respectively. Finally, tramadol was present in lake Mälaren up to 18 ng/L, showing occurrences also in Swedish aquatic ecosystems [35].

Citalopram is an antidepressant of the SSRI type. Swedish researchers have shown partially inhibited feeding [54] and long-lasting behavioral effects after exposure during development of three-spine stickleback (*Gasterosteus aculeatus*) to relevant environmental concentrations [55]. The Scanian WWTPs had concentrations between 80 and 217 ng/L, with an average concentration of 128 ng/L. This was close to data from Stockholm Vatten for Henriksdal and Bromma WWTPs, with concentrations of 196 ng/L (RSD = 44%) and 140 ng/L (RSD = 56%), respectively [24]. Slightly higher concentrations were found in National Screening [34] ranging between 170 and 480 ng/L. In the EU Monitoring, the detection frequency was 83%, maximum reported concentration 189 ng/L and average concentration 34 ng/L [39]. Overall, the Scanian concentrations were on par with previously reported concentrations. The highest concentration quantified in Swedish lake Mälaren was 4.1 ng/L [35].

Fluconazole is an antifungal medication, and the concentrations varied between 3 and 105 ng/L, with an average concentration of 48 ng/L. An analysis of fluconazole was not performed by Stockholm Vatten [24]. National Screening, however, reported much higher concentrations ranging from 72 to 1100 ng/L [34]. What might cause this large difference is not known. The effluents from 11 Swedish WWTPs showed a detection frequency of 97% with maximum and average concentrations of 170 and 60 ng/L, respectively [47] which is very close to our findings. Previous Swedish studies of five WWTPs had similar concentrations ranging between <LOQ and 140 ng/L [56]. The EU Monitoring [39] had a detection frequency of 98% and maximum and average concentrations of 598 and 108 ng/L, respectively, which is somewhere between the Scanian values and the National Screening values.

Sertraline is also an SSRI-type antidepressant, as citalopram above. Swedish researchers have studied the behavior of freshwater snails (*Radix balthica*) exposed to the pharmaceutical, observing a general lack of effects on the snail’s activity [57]. In contrast, the same researchers found that the fish species Eurasian perch (*Perca fluviatilis*) presented an exposure-dependent decrease in feeding habits with increasing sertraline concentrations at a µg/L level [58]. The concentrations in the Scanian WWTPs varied in the interval 4–47 ng/L. The average concentration of 24 ng/L was similar to Henriksdal and Bromma, with concentrations of 26 ng/L (RSD = 59%) and 21 ng/L (RSD = 72%), respectively, Stockholm Vatten [24]. In National Screening [34], concentrations were between 0 and 32 ng/L, while in the EU Monitoring [39] the reported maximum and average concentrations were 38 and 2 ng/L, respectively, but with a detection frequency of only 12%. The sertraline concentrations in Scania are close to those observed in other studies. Sertraline could not be identified in lake Mälaren, Sweden [35].

##### Hormones

Two hormones were analyzed, namely estrone (a natural hormone) and levonorgestrel (a synthetic hormone), as shown in Figure 3 and Supplementary Materials, Table S2.

Estrone and other estrogenic substances in WWTP effluents have since long been known to induce estrogenic effects in fish [59]. In Scania, estrone occurred in low concentrations in outlet wastewater between 1 and 18 ng/L, except for Ormanäs WWTP, at 63 ng/L. The average concentration, with and without the Ormanäs value, was 13 and 6 ng/L, respectively. This is similar to Stockholm Vatten [24], where estrone analyses were performed as discrete “special analyses” and found concentrations of 4.2 ng/L (RSD = 89%) and 0.5 ng/L (RSD = 46%) at Henriksdal and Bromma, respectively. Estrone was not included in the National Screening [34], while in the EU Monitoring [39] it was analyzed by a multi-compound hormone screening method. However, no hormones were detected in any of the samples above the LOQ of 10 ng/L.

Levonorgestrel has been investigated by Swedish researchers and shown to disrupts the seasonal breeding cycle in male three-spine stickleback (*Gasterosteus aculeatus*) at ng/L levels [60]. Levonorgestrel could not be detected in any Scanian wastewaters, while Stockholm Vatten [24] did not analyze it. No levonorgestrel could be quantified in the water samples in National Screening [34], while it was not included in the EU Monitoring [39].

##### Ketoconazole, Zolpidem and Methotrexate

These pharmaceuticals are all present in low concentrations (Figure 3 and Supplementary Materials, Table S2).

Ketoconazole is an antifungal compound not quantified in any of the Scanian WWTPs. Stockholm Vatten [24] found 8 ng/L (RSD = 77%) at Henriksdal and 9 ng/L (RSD = 81%) at Bromma WWTP. In National Screening [34], it was only be found in one of 12 samples, but with the remarkably high concentration of 120 ng/L. In the 11 Swedish WWTPs investigated, very high levels of ketoconazole were found in sludge, with an average concentration of 2835 ng/g dry weight and a detection frequency of 85%. Consequently, it binds to sludge [47]. In the same study, the effluent sample had maximum and average concentrations of 41 and 36 ng/L, respectively, which is slightly higher than previous Swedish studies. Ketoconazole was also recently quantified by us in sludge, showing concentrations as high as 3009 ng/kg [48]. Ketoconazole was not analyzed in the EU Monitoring [39].

Zolpidem is administered for difficulties with sleeping. The average concentration was 3 ng/L, ranging from 1 to 4 ng/L. The concentrations at Henriksdal and Bromma were 5.1 ng/L (RSD = 46%) and 4.8 ng/L (RSD = 55%), respectively, according to Stockholm Vatten [24]. In National Screening [34], zolpidem concentrations varied between 3 and 41 ng/L. In the EU Monitoring [39], it had a detection frequency of 58%, with maximum and average concentrations of 43 and 1.5 ng/L, respectively, which is relatively close to the Swedish studies.

Methotrexate is used against rheumatic and inflammatory diseases, and in cancer treatment. It was not detected in any of the Scanian WWTP outlet water samples. No analysis of methotrexate was performed in the two previous Swedish studies by Stockholm Vatten [24] and National Screening [34]. It was not analyzed in the EU Monitoring, either [39].

#### 2.2.2. Chemical Emissions of Pharmaceuticals from Eight WWTPs in Kilograms

**Figure 7:**
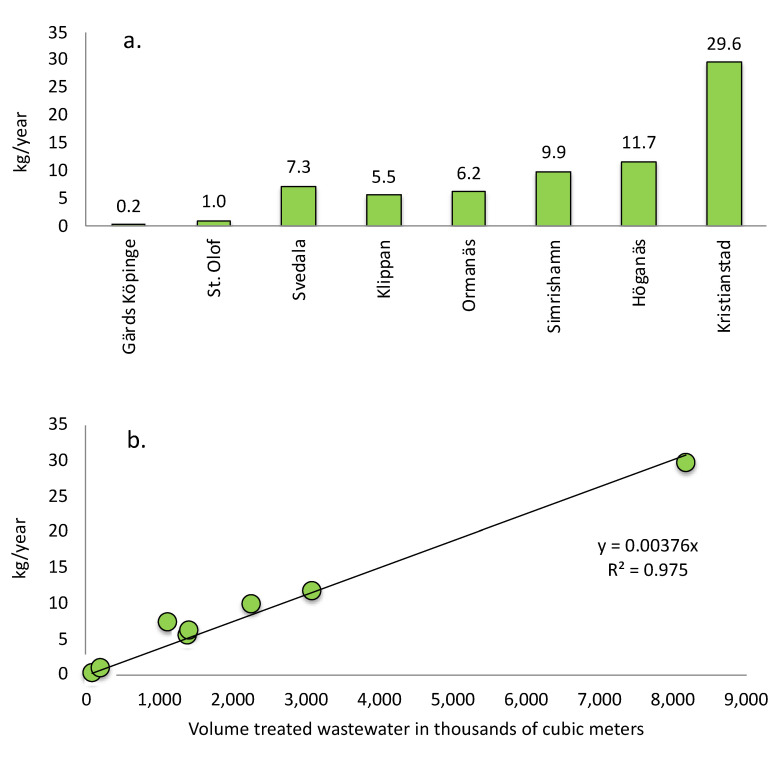
(**a**) Total emission in kg/year of the 21 pharmaceuticals in the 8 Scanian WWTPs. (**b**) Relationship between the emitted amount of pharmaceuticals as the sum of 21 pharmaceuticals in kg/year (y-axis) and the treated wastewater volume in thousands of cubic meters per year. In both panels, the order of the WWTPs is based on size according to Figure 2b.

The basic parameters of the WWTPs comprised the annual volume of treated wastewater (Supplementary Materials, Table S1a). By multiplying this volume with the outlet concentrations of each pharmaceutical at each WWTP (Supplementary Materials, Table S2), an estimate of the annually emitted amounts of pharmaceuticals in grams could be calculated (Supplementary Materials, Table S4). Thereafter the pharmaceutical emissions from each WWTP in kilograms could be estimated. The results are seen in Figure 7a.

The emissions from the eight WWTPs added up to a total of 71,409 which equals ca. 71 kg. These 71 kg only includes those 21 pharmaceuticals analysed. The smallest WWTP in Gärds Köpinge released only 0.2 kg while the largest WWTP in Kristianstads released as much as 30 kg. By relating the released amount of drugs per year (kg/year) to the treated volume of water in thousands of cubic meters (m^3^) the relationship between the volume wastewater and the emitted amount of pharmaceuticals could be estimated (Figure 7b). From this, it could be seen that, when a WWTP in Scania releases 1 million cubic meter (1,000,000 m^3^) of treated wastewater, roughly 3.76 kg ≈ 4 kg of the 21 pharmaceuticals passes through into the recipient simultaneously.

### 2.3. Estimate of Total Pharmaceutical Emissions in All of Scania Based on Open Data of Amount Treated Wastewater from Scanian WWTP Operators

**Figure 8:**
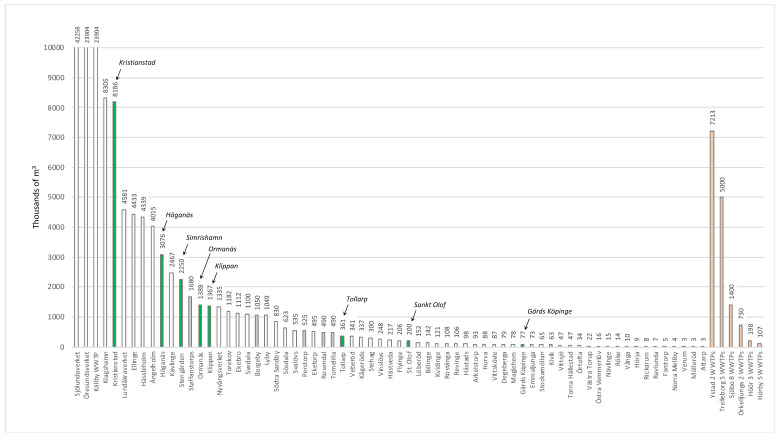
Yearly volume treated wastewater in thousands of m^3^ in County Scania. For explanations, see text.

Scania has 33 municipalities and a population of 1,322,193 persons (as of 2016) [61]. The WWTP operators most often release data on the total volume of treated wastewater. As part of this study, a large effort was put forth to identify as many of these data as possible to estimate the total amount of treated wastewater in Scania. An overview of the 92 identified WWTPs and their yearly volume of treated wastewater is shown in Figure 8, where the great diversity in size of WWTPs becomes clear. The figure also shows that the eight WWTPs in this study (marked in green) covers this diversity. Not all data could be identified for all 92 WWTPs, and, in such cases, the volume of wastewater was estimated (as discussed below). These WWTPs were, however, few (marked in gray in Figure 8). In some cases, several WWTPs were reported as a sum of treated wastewater (indicated to the right of Figure 8 in light red). Summing all reported volumes gave a total volume of 152,887 thousand m^3^ of wastewater. This can be compared to the total treated volume of wastewater in Sweden, which, in 2016, was 1,078,652 thousand m^3^. Consequently, Scania has ca. 14.2% of the wastewater in Sweden. This is realistic, considering that the population of Scania was 1,322,193 persons in 2016, compared to Sweden’s total population of 9,995,153 the same year [61], corresponding to 13.2% of the total Swedish population. The population of Scania is not evenly distributed, and especially the western side of Scania has several larger cities. In Southern and Eastern Scania, the size of the population is slightly lower with a few small- and medium-sized cities. The identified treated wastewater volumes were grouped into six different geographical regions: Northwest, Southwest, South, Southeast, Northeast and Central Scania.

#### 2.3.1. Northwestern Scania

Nordvästra Skånes Vatten och Avlopp AB (NSVA) is the organization that treats a majority of the wastewater in this area in six municipalities: Bjuv, Båstad, Helsingborg, Landskrona, Svalöv and Åstorp. In total, 10 WWTPs are operated with differing size, according to the available environmental reports (Supplementary Materials, Table S5). The total volume is 33,710,670 m^3^, resulting in 126.8 kg. Additionally, Höganäs Municipality and its WWTP releases 11.7 kg according to our study, while the Klippan Municipality WWTP discharges 5.6 kg. The WWTP in Ängelholm Municipality treats around 11,000 m^3^ of wastewater/day, according to their information, corresponding to 4,015,000 m^3^ wastewater/year resulting in 15.1 kg. The total emission is estimated to be as follows (Equation (1)):

126.8 + 11.7 + 5.6 + 15.1 = 159.2 kg ≈ 160 kg/year(1)

#### 2.3.2. Southwestern Scania

The largest organization in this part is VA SYD AB, which, all together, treats wastewater from more than 500,000 people. One of Sweden’s largest WWTPs is Sjölundaverket, which treats wastewater from the City of Malmö and Burlöv, Lomma, Staffanstorp and Svedala Municipalities. Klagshamn WWTP receives wastewater from some areas of Malmö City and Vellinge Municipality. Additionally, VA SYD operates several other WWTPs as seen in Supplementary Materials Table S5, with a total volume of 68,694,617 m^3^ resulting in 258.3 kg. For Borgeby WWTP in Lomma Municipality the exact volume is not known, but the WWTP is dimensioned for 15,000 persons. Based on similar-sized WWTPs this would give roughly 1,050,000 m^3^ of wastewater and 3.9 kg. Likewise, Staffanstorp Municipality treats wastewater from 14,000 persons, corresponding to 980,000 m^3^ wastewater and 3.7 kg. For Kävlinge Municipality the stated volume treated wastewater was 6758 m^3^/day, corresponding to 2,466,670 m^3^/year and 9.3 kg. In addition, Svedala Municipality discharges 7.3 kg, according to our own study. The total emission is estimated to as follows (Equation (2)):

258.3 + 3.9 + 3.7 + 9.3 + 7.3 = 282.5 kg ≈ 283 kg/year(2)

#### 2.3.3. Southern Scania

Here Trelleborg Municipality operates five WWTPs in Trelleborg, Smygehamn, Västra Alstad, Sjörup and Grönalund, which annually treat at total of 5,000,000 m^3^ wastewater, corresponding to 18.8 kg. Ystad WWTP treats wastewater from Ystad and Skurup Municipality, in total 7,212,600 m^3^ of wastewater/year, resulting in 27.1 kg. The total emission is estimated to be as follows (Equation (3)):

18.8 + 27.1 = 45.9 kg ≈ 46 kg/year(3)

#### 2.3.4. Southeastern Scania

Simrishamn Municipality has >19,000 residents; Stengården WWTP is the municipality’s largest WWTP and was included in this study, with a discharge of 9.9 kg. Simrishamn Municipality operates a few smaller WWTPs: Kivik, Sankt Olof, Östra Vemmerlöv, and Ravlunda. The treated volume wastewater was not stated, but their dimensions were 3000, 1000, 250 and 140 PE. Sankt Olof treatment plant was measured in this study to discharge 1.0 kg. The additional three WWTPs would give 3400 PE and a volume of 238,000 m^3^ wastewater and 0.9 kg pharmaceuticals. Tomelilla Municipality has Rosendal WWTP which treats water from 7000 persons, corresponding to 490,000 m^3^ and 1.8 kg. Sjöbo Municipality has ca. 19,000 residents, and the treatment of wastewater is performed at Sjöbo WWTP and in seven smaller WWTPs. In total, 1,400,000 m^3^ of wastewater is treated yearly, resulting in 5.3 kg. The total emission is estimated to be as follows (Equation (4)):

9.9 + 1.0 + 0.9 + 1.8 + 5.3 = 18.9 kg ≈ 19 kg/year(4)

#### 2.3.5. Northeastern Scania

Kristianstad Municipality has the largest WWTP, which was also included in this study, discharging 29.6 kg. Kristianstad Municipality operates 11 other WWTPs, where Gärds Köpinge was also part of this study, with 0.2 kg. The additional seven WWTPs are Arkelstorp, 92,501 m^3^; Vittskövle, 86,989 m^3^; Degeberga, 79,000 m^3^; Maglehem, 78,283 m^3^; Vånga, 9790 m^3^; and Rickarum, 8264 m^3^, with a total volume of 431,365 m^3^ and 1.6 kg pharmaceuticals. For the two smallest WWTPs, no volume could be found. The municipalities of Bromölla, Östra Göinge and Osby are located north of Kristianstad, and wastewater treatment is performed by Skåne Blekinge Vattentjänst AB (SBVT). The volume of treated water in Bromölla Municipality is 1,270,500 m^3^/year, corresponding to 4.8 kg. Östra Göinge Municipality has a number of WWTPs in Knislinge, Broby, Sibbhult, Immeln, Östanå, Boalt and Kräbbleboda, which treats 1,600,000 m^3^ wastewater per year, corresponding to 6.0 kg. Five WWTPs are operated in Osby Municipality: Osby town, Lönsboda, Killeberg, Hökön and Visseltofta. These correspond to 1,600,000 m^3^/year and 6.0 kg of pharmaceuticals. The total emission is estimated to be as follows (Equation (5)):

29.6 + 0.2 + 1.6 + 4.8 + 6.0 + 6.0 = 48.2 kg ≈ 48 kg/year(5)

#### 2.3.6. Central Scania

Örkelljunga Municipality has around 10,000 residents and two WWTPs in Skånes Fagerhult and Örkelljunga. The municipality stated that, every day, between 2000 and 5000 m^3^ of wastewater is treated, which gives a minimum of 730,000 m^3^ of wastewater treated yearly, corresponding to 2.7 kg of pharmaceuticals. Perstorp Municipality has 7000 residents, but no information about WWTPs could be found, so they were excluded from calculations. Hässleholm Municipality has 52,000 residents, and the wastewater is treated by Hässleholms Vatten AB. The treatment plants are listed in Supplementary Materials, Table S5. Hässleholm Vatten treats a total volume of 5,623,878 m^3^/year, corresponding to 21.1 kg. Höör and Hörby Municipalities have a total of 32,000 residents. Wastewater treatment is performed by the organization Mittskåne Vatten, which operates 10 WWTPs. Hörby Municipality has six WWTPs, and Höör Municipality consequently has four. In our study, Ormanäs WWTP was included, discharging 6.2 kg. Apart from this, 1,048,604 m^3^ wastewater is treated in Lyby WWTP, releasing 3.9 kg. The additional eight WWTPs together generate 304,997 m^3^, corresponding to 1.1 kg. The total emission is estimated to be as follows (Equation (6)):

2.7 + 21.1 + 6.2 + 3.9 + 1.1 = 35.0 kg ≈ 35 kg/year(6)

#### 2.3.7. Total Estimated Emission of Pharmaceuticals in Scania

The calculated chemical burdens above are estimates that build on information available from the municipalities. Additionally, some WWTPs are not part of the calculations, as information was lacking. Furthermore, in some cases, the WWTPs must brim their wastewater; thereby, it is released untreated directly to the recipient, thus increasing the burden. Furthermore, the study does not take into account private sewage systems, so-called on-site sewage facilities (OSSFs), which are known to be less efficient and release an unknown number of pharmaceuticals [62]. Around 10% of the Swedish population (1 million persons) is connected to such OSSFs. One should also consider that only a very limited number of pharmaceuticals, out of several hundred active medications on the market, were included in this study. Nonetheless, this study adds up to the following (Equation (7)):

159.2 + 282.5 + 45.9 + 18.9 + 48.2 +35.0 = 589.7 kg ≈ 590 kg/year(7)

This total load in Scania and the distribution between the various areas are illustrated in Supplementary Materials, Figure S4. Still, it is not unlikely that the total pharmaceutical load to Scanian waters is many times larger, meaning one or several tons yearly.

### 2.4. Occurrence of Pharmaceuticals in Scanian Streams and Lakes Downstream WWTPs

**Figure 9:**
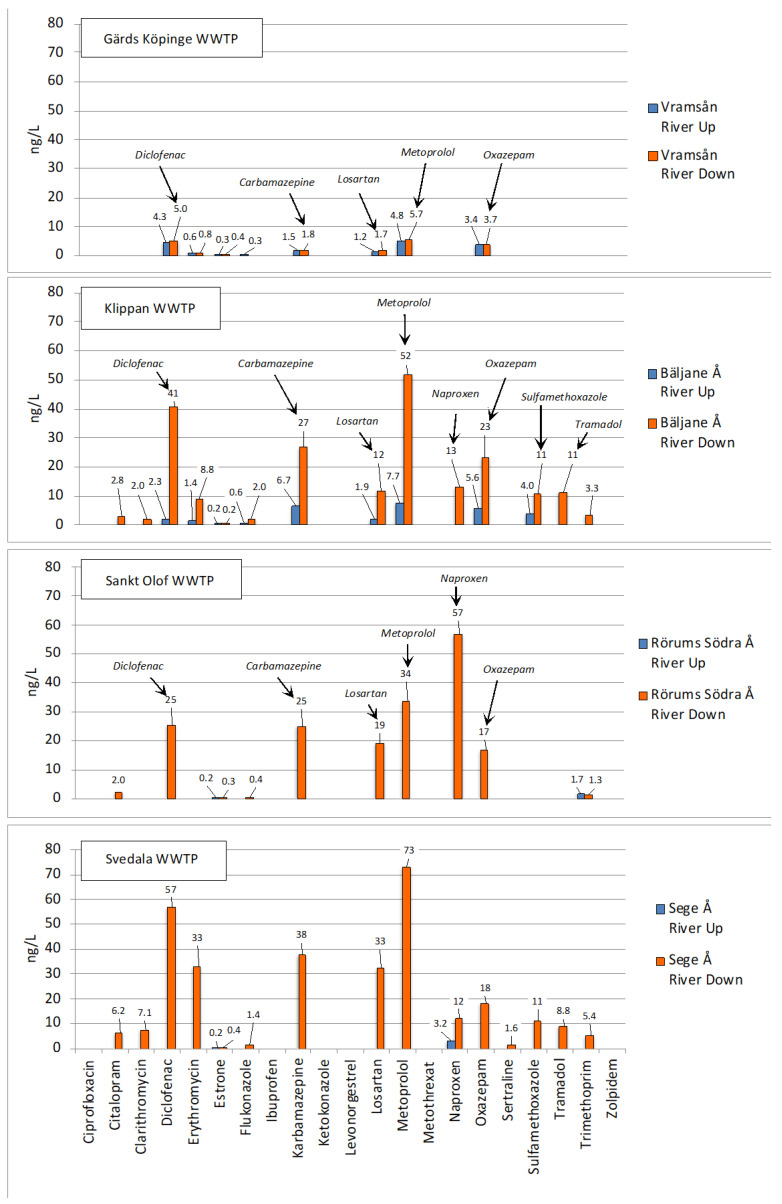
Measured concentrations of pharmaceuticals in the 4 investigated rivers, upstream (Up) and downstream (Down) of the WWTPs.

A critical topic is whether the concentrations of drugs in the recipient are increasing downstream the WWTPs. For study this, water sampling was performed in the recipient both upstream and downstream of the WWTPs. The sampling of these environmental waters depended on the geographical placement of the various WWTPs. The results of the analyses are seen in Supplementary Materials, Table S6 and visualized for the four rivers in Figure 9. Based on this information, the pharmaceutical occurrence could be compared between different types of recipients.

#### 2.4.1. Gärds Köpinge WWTP and Vramsån River

Gärds Köpinge WWTP discharges ca. 8.75 m^3^/h or 0.0024 m^3^/s of wastewater into the Vramsån River which has an annual flow of approximately 4 m^3^/s [63]. The sampling sites in Vramsån river are seen in Supplementary Materials, Figure S5. The results show that this WWTP has a limited impact on the concentrations of pharmaceuticals in the river (Figure 9), since they basically are at the same low level before and after the WWTP, and always less than 6 ng/L. This can be explained by the tiny contribution from the WWTP to the total river flow. The ratio between the average annual flow (m^3^/s) of the river and the daily flow from the WWTP (m^3^/s) gives a value of 1667 (4/0.0024), revealing extensive dilution. However, it is noteworthy that the concentrations upstream Gärds Köpinge WWTP for some pharmaceuticals (diclofenac, carbamazepine, losartan, metoprolol and oxazepam) also occur at a few ng/L (Figure 9). The reason for this might be that the larger WWTP in Tollarp is located only a few kilometers upstream. Studies are underway to determine the contribution from Tollarp WWTP to the chemical load of the Vramsån River.

#### 2.4.2. Klippan WWTP and Bäljane Å River

Klippan WWTP releases 156 m^3^/h, or 0.043 m^3^/s, wastewater into the Bäljane Å river, which has an average annual flow of 2.4 m^3^/s [64]. The average dilution factor in the river has been reported to be 40-fold; however, in extremely low flow periods, it may drop to 2-fold, meaning that, at times, the river can comprise one-third of the treated wastewater [64]. The sampling points are shown in Supplementary Materials, Figure S5. From Figure 9, it can be seen that the concentrations downstream Klippan WWTP are higher than upstream: diclofenac, 41 vs. 2.3 ng/L; carbamazepine, 27 vs. 6.7 ng/L; losartan, 12 vs. 1.9 ng/L; metoprolol, 52 vs. 7.7 ng/L; naproxen, 13 ng/L vs. <LOQ; oxazepam, 23 vs. 5.6 ng/L; sulfamethoxazole, 11 vs. 4.0 ng/L; and tramadol, 11 ng/L vs. <LOQ. The Bäljane Å river runs into the Rönne Å river, which thereafter feeds into the sea at Skälderviken Bay on the western coast of Scania. In previous studies, seawater was sampled from this coast in a Baltic Sea Expedition [41]. Of the 92 pharmaceuticals analyzed, carbamazepine was found in all samples at concentrations between 1.2 and 2.4 ng/L, fluconazole at 1.0 ng/L (1 sample), ketoconazole at 5.5 ng/L (one sample) and metoprolol at 1.0 and 2.3 ng/L (two samples). Accordingly, there is some agreement between those pharmaceuticals found at the highest concentration in the Bäljane Å River and those quantified in the western coast of the Baltic sea. By dividing the concentrations in the river downstream the WWTP with concentrations upstream the WWTP it was shown that the downstream concentrations are roughly six times higher at Klippan WWTP for diclofenac, erythromycin, fluconazole, carbamazepine, losartan, metoprolol, oxazepam and sulfamethoxazole. Likewise, by dividing the outlet concentrations in the WWTP with the downstream river concentrations, it is shown that the outlet concentrations are 22 times higher on average. This is in relatively good agreement with the average dilution factor of 40 [64]. The ratio between the average annual flow of the river and the daily flow of wastewater of the WWTP yields a value of 56 (2.4/0.043), which is also fairly close to 40. Overall, this indicates that dilution plays a major role in the reduction of pharmaceuticals in the river, though not as large as in the Vramsån river above.

#### 2.4.3. Sankt Olof WWTP and Rörums Södra Å River

Sankt Olof WWTP releases its water into Rörums Södra Å river. Flow data were not available; however, a realistic estimate would be 20 m^3^/h or 0.0056 m^3^/s. The Rörums Södra Å river has a reported flow of 0.4 m^3^/s [65]. The sampling sites are shown in Supplementary Materials Figure S5, while the concentrations of pharmaceuticals are shown in Figure 9, revealing elevated concentrations downstream Sankt Olof WWTP. Six pharmaceuticals exceed 10 ng/L: diclofenac, 25 ng/L; carbamazepine, 25 ng/L; losartan, 19 ng/L; metoprolol, 34 ng/L; naproxen, 57 ng/L; and oxazepam, 17 ng/L. The river runs eastwards and feeds directly into Hanöbukten bay of the Baltic Sea. In the Baltic Sea Expedition [41], no samples were taken directly in the Hanöbukten Bay, but two samples were taken in the Southern Baltic proper, where carbamazepine was quantified in two samples at 2.1 and 3.3 ng/L, and fluconazole in one sample at 1.9 ng/L. Looking deeper into the river data, a few things can be noted in concerning the observations made for Gärds Köpinge WWTP in the Vramsån river and Klippan WWTP in the Bäljane Å river (above). The size of Gärds Köpinge WWTP is about the same as Sankt Olof WWTP, even so higher concentrations of pharmaceuticals was not observed in the Vramsån river as dilution was higher. Calculating the relationship between the annual average river flows and the daily wastewater flows at the WWTPs in Gärds Köpinge and Sankt Olof generated values of 1667 (above) and 71 (0.4/0.0056), respectively. Consequently, the dilution of wastewater in the Vramsån river is 23 times larger than in the Rörums Södra Å river. This results in considerably lower concentrations in the Vramsån river. Turning to Klippan WWTP, it can be seen that the relation between the flow of wastewater per day and the Bäljane Å river annual average flow is 56 (above). This relation is similar to that observed in the Rörums Södra Å river, with a corresponding value of 71. The similarities in concentrations in the two rivers is therefore not startling (Figure 9). By looking at upstream concentration data in the Rörums Södra Å river, we see that Sankt Olof WWTP seems to be the main pharmaceuticals source in this river.

#### 2.4.4. Svedala WWTP and Sege Å River

Svedala WWTP releases 125 m^3^/h or 0.035 m^3^/s wastewater into the Sege Å River. The average river flow has been reported to 2.7 m^3^/s [66], and the sampling sites in are shown in Supplementary Materials, Figure S5. The concentrations of pharmaceuticals downstream Svedala WWTP are shown in Figure 9 which reveals an increased occurrence. In total, eight pharmaceuticals had concentrations exceeding 10 ng/L: diclofenac, 57 ng/L; erythromycin, 33 ng/L; carbamazepine, 38 ng/L; losartan, 33 ng/L; metoprolol, 73 ng/L; naproxen, 12 ng/L; oxazepam, 18 ng/L; and sulfamethoxazole, 11 ng/L. The river feeds into the Southern Lommabukten bay of the western coast of Scania (just as the Bäljane Å river above), and correspondingly there is some correlation between the compounds released into the Sege Å river and those identified on the western coast, i.e., carbamazepine, fluconazole, ketoconazole and metoprolol [41]. The ratio between the average annual river flow and the daily wastewater for the WWTP results in a value of 77 (2.7/0.035). This value is resembling both the value 56 for Bäljane Å river and the value 71 for Rörums Södra Å river at. It is therefore reasonable that the concentrations downstream Svedala WWTP in the Sege Å river are similar to those reported in the Bäljane Å river, as well as the Rörums Södra Å river above.

#### 2.4.5. Kristianstad WWTP and Hammarsjön Lake

**Figure 10:**
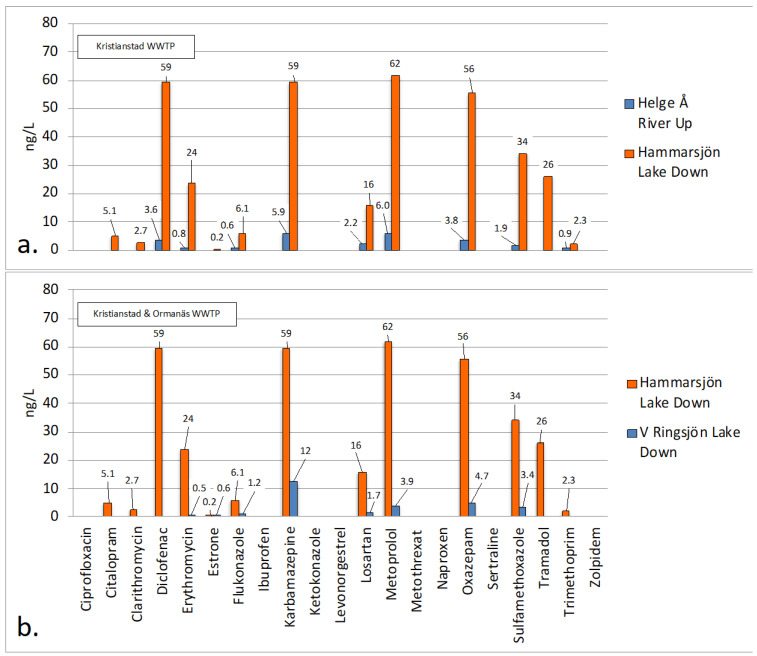
(**a**) Measured concentrations of pharmaceuticals in the Helge Å river upstream and the Hammarsjön lake downstream of Kristianstad WWTP. (**b**) Comparison of measured concentrations of pharmaceuticals in the Västra Ringsjön lake downstream Ormanäs WWTP and the Hammarsjön lake downstream Kristianstad WWTP.

Kristianstad WWTP releases 958 m^3^/h or 0.27 m^3^/s wastewater into the Hammarsjön lake with a volume of 782,000 m^3^. The upstream sampling point is situated in the Helge Å River (Supplementary Materials, Figure S6), and has an approximate flow of 39 m^3^/s [63]. The Helge Å river enters the Hammarsjön lake in its northwestern corner. The location of the sampling point downstream Kristianstad WWTP is ca. 2 km from the WWTP at a place named “Ekenabben” situated in the northeastern corner of the Hammarsjön lake. From Figure 10a it is clear that the occurrence of pharmaceuticals downstream at “Ekenabben” are higher than those found at the upstream sampling point in the river. On average the lake concentrations are 13 times higher than the river concentrations upstream the WWTP. Similarly, Kristianstad WWTP outlet concentrations are 11 times higher (on average) than concentrations downstream the WWTP in the Hammarsjön lake. Consequently, the pharmaceutical concentrations are diluted 11 times in the Hammarsjön lake before reaching “Ekenabben” inlet. Simultaneously, a minor calculation reveals that the treated wastewater contributes quite substantially to the total lake volume considering that the volume of the lake is ca. 782,000 m^3^ and Kristianstad WWTP releases 958 m^3^ water per hour. Through the following basic calculation, 782,000 m^3^/958 m^3^/h = 816 h = 34 days, it can be seen that, in only 1 month, the released volume of treated wastewater equals the entire volume of the Hammarsjön lake. Despite this, the lake concentrations are still not extremely high, and this probably can be explained by the fact that the Helge Å river flows through the lake at a rate of ca. 39 m^3^/s = 140,400 m^3^/h. It can therefore be estimated that the Helge Å river renews the lake water in ca. 6 h. No identification of the flow profile of the lake water has been found, but even though the flow of water through the lake is high, rather high concentrations are still observed at the inlet “Ekenabben”. This is probably because this is a more stagnant part of the lake.

#### 2.4.6. Ormanäs WWTP and Västra Ringsjön Lake

Ormanäs WWTP releases 184 m^3^/h, or 0.051 m^3^/s, wastewater into the Västra Ringsjön lake. The downstream sampling point is shown in Supplementary Materials Figure S6, while no upstream sampling point could be located. The exact position of the outlet of the wastewater in the lake was not known, but most likely some distance from the shore. Even so, lake water was sampled 2 m from the shore in a southwesterly direction from the WWTP. The volume of the Västra Ringsjön lake is ca. 39,110,000 m^3^ and a comparison of the concentrations of pharmaceuticals in the Hammarsjön lake and the Västra Ringsjön lake is shown in Figure 10b. The Hammarsjön lake had concentrations that were 13 times higher than the Västra Ringsjön lake. Even though the point of release of wastewater in the Västra Ringsjön lake is not exactly known and the lake currents were not identified, we can still estimate that the Västra Ringsjön lake volume is around 50 times greater than the Hammarsjön lake volume. Consequently, the dilution is thereby much bigger. Additionally, Ormanäs WWTP only makes a minor contribution of water compared to the total volume of the lake. Through the following simple calculation, 39,110,000 (m^3^)/184 (m^3^/h) = 212,554 h = 8856 days, it takes 24 years until the discharged volume treated wastewater equals the water volume of the Västra Ringsjön lake. It should be noted though, that the Västra Ringsjön lake lacks the flow of a river as large as the Helge Å river and therefore the turnover of water in the Västra Ringsjön lake is therefore most likely not as high.

#### 2.4.7. Höganäs WWTP and Öresund and Simrishamn WWTP and the Baltic Sea

Höganäs WWTP and Simrishamn WWTP discharge their wastewater into a sea environment and their location are shown in Supplementary Materials Figure S7. For both WWTPs the volume of the recipient is unknown and no downstream samples were taken. The recipient Öresund (western coast) is likely not as sensitive as the Baltic Sea (eastern coast), since the latter is a closed brackish water sea. At the same time, the released amount of pharmaceuticals (kg) into Öresund is much larger than into the Baltic Sea (Supplementary Materials Figure S4). Based on the fact that fresh water is becoming a limited resource during the summer period it might be worthwhile to consider to stop releasing the wastewater into the salty water of the sea, since the costs of turning it back into fresh water is very costly. Additionally, there is a shortage of fresh water in the summer season in many parts of Sweden, and therefore increased reuse and circulation of fresh water may be a way to improve water deficiency in these areas.

## 3. Materials and Methods

### 3.1. Questionnaire, Wastewater Treatment Plants (WWTPs) and Recipients

Eight WWTPs in Scania (the most southern county in Sweden) were included in the study, covering several different scenarios. This constituted a suitable basis for model studies of pharmaceutical emissions from the roughly 90 WWTPs in Scania. All participating WWTPs were given a questionnaire and asked to provide basic information about the WWTPs, such as size and volumes. The answers to these questions are shown in Supplementary Materials Table S1. The 8 WWTPs chosen are spread out geographically all over Scania and discharge their wastewater in several differing recipients, as seen in Figure 2a.

In Northwest Höganäs, WWTP discharges directly into the salty water of the Öresund coastal sea area, Kattegatt. In inland Northwest Scania, Klippan WWTP has a point of discharge in the Bäljane Å river that continues to Rönne Å river and thereafter reaches Ängelholm and the Skälderviken bay of the western coast of Scania. In Southwest Scania, Svedala WWTP has a point of discharge in the Sege Å river. This river ends in the Southern Lommabukten bay on the western coast. In Central Scania, Ormanäs WWTP discharges wastewater in the northern part of the Västra Ringsjön lake. In Northeastern Scania, Kristianstad WWTP and Gärds Köpinge WWTP were both included. Both are situated in the UNESCO Biosphere Reserve Area Kristianstads Vattenrike [67] and connected to the basin of the Helge Å river, as described in more detail in our previous paper [68]. Kristianstad WWTP discharges wastewater into a 1500 m long canal, that ends in the Hammarsjön lake. The Helge Å river is the largest river in Scania, and both the inlet and outlet of the river are located in the Hammarsjön lake. The point of discharge of Gärds Köpinge WWTP is in the Vramsån river, which runs into the Helge Å river. The Helge Å river thereafter continues on eastwards to Hanöbukten bay (Baltic Sea with brackish water), with its largest exit in Gropahålet (close to the village Yngsjö) and a minor exit in Åhus harbor. Sampling was also performed at Sankt Olof WWTP and Simrishamn WWTP, both located in the southeast corner of Scania. Sankt Olof WWTP releases its wastewater into the Rörums Södra Å river, which continues east and ends in the Hanöbukten Bay north of Vik. Simrishamn WWTP discharges its wastewater directly into the Hanöbukten bay.

### 3.2. Sampling and Analysis

In spring 2017, contact was taken with the WWTP organizations before sampling. The actual sampling campaign was performed through visits at the 8 WWTPs on the 4/5 April 2017. All samples underwent chemical analysis at Kristianstad University during April 2017. All sampling was conducted in collaboration with the WWTP staff. All samples at the WWTP were grab samples taken in 100 mL HDPE bottles. Surface-water samples from rivers and lakes were all collected by the authors as grab samples, using 500 mL HDPE bottles. Samples were taken 0.2 m below surface for all rivers and lake samples. All sample types were kept frozen at −18 °C until analysis. For pharmaceutical determination, 50 and 500 mL of the collected sample volume were extracted for wastewaters and surface waters, respectively. For extraction, HLB cartridges (hydrophilic–lipophilic balance 200 mg sorbent, 6 mL cartridge) were purchased from Waters Oasis (Milford, MA, USA). In this project, a robust and flexible method developed at Kristianstad University, Sweden, was used [30,31]. The method has been validated according to a method established by the United States Environmental Protection Agency (US EPA) in 2007 for analysis of pharmaceuticals and personal hygiene products in water, soil, sediment and biomaterial, using HPLC/MS/MS [69]. The method has also been validated in a recent intercalibration exercise funded by the Swedish Agency for Marine and Water Management, including 5 well-established laboratories (4 Swedish and 1 Danish), demonstrating good overall performance [53]. For analysis, a Waters Acquity UPLC H-Class with the following components was used: a Quaternary Solvent Manager (QSM), a Sample Manager with Flow-Through Needle (SM-FTN) and a Column Manager (CM), enabling fast column switching (Waters, Milford, MA, USA). The chromatographic column was a Waters Acquity UPLC BEH C18 column (2.1 mmID × 50 mm, 1.8 µm). For final detection, a Xevo TQ-S™ triple quadrupole mass spectrometer (Waters Micromass, Manchester, UK) equipped with a Z-spray electro-spray interface was used.

## 4. Conclusions

The concentrations of pharmaceuticals released by the eight WWTPs in County Scania are, in general, on par with those reported in previous Swedish monitoring studies. There is also some agreement with previous European screening programs. Of the 21 analyzed substances, metoprolol and diclofenac have the highest average outlet concentrations of 946 and 680 ng/L, respectively. This is followed by carbamazepine, losartan, naproxen and oxazepam, all of which are above 250 ng/L.

A separate investigation on seasonal variation in outlet concentration from the largest WWTP in Kristianstad showed a peak in January and a dip in July, but still with a consistent flow of pharmaceuticals to the recipient all year around.

Despite the fact that the concentrations are low (ng/L to µg/L levels), the total chemical burden from these eight WWTPs is substantial on a yearly basis and adds up to 71 kg, as a consequence of the large total volumes of treated wastewater. The total treated wastewater volume for the >90 WWTPs in Scania was estimated to be 152,887,000 m^3^, which is ca. 14% of the total Swedish wastewater volume. Based on a correlation between the volume of treated wastewater and the total amount released of the 21 pharmaceuticals, an estimate of the total chemical burden to the Scanian aquatic systems could be performed. This showed a total release of 590 kg/year.

An analysis of river samples upstream and downstream the WWTPs showed that the dilution factor of the WWTP wastewater flow in the receiving river flow is central for the concentrations found downstream of WWTPs. In short, small WWTPs in large rivers causes less concern. Still, the need for implementation of advanced treatment should be judged on a case-by-case basis, as there might be specific ecological values that need protection also from minor discharges. For example, the Vramsån river has fine specimens of the very rare freshwater pearl mussel (*Margaritifera margaritifera*) [70] which can live for more than 250 years [71]. Being exposed to even minute amounts of contaminants for centuries may cause unknown effects, especially considering that these mussels are sensitive to environmental pollution [72].

In summary, regional monitoring studies will aid in prioritizing where the need for advanced treatment is highest, as requested by the Swedish EPA [28]. Even a few measurements can make a large difference in dialogue with politicians and stakeholders. Considering the large financial undertakings associated with construction of modern treatment facilities that should last for decades, the time and resources spent on a sampling campaign is quite insignificant. It will provide essential information in taking knowledge-based decisions that will save time and resources and, at the same time, make sure that the investments made will maximize the protection of our aquatic ecosystems.

## Data Availability

The chromatograms of the analyses are stored on a server at Kristianstad University, Sweden.

## Supplementary Materials

The following supporting information can be downloaded. Figure S1: Outlet concentrations of diclofenac and metoprolol. Figure S2: Outlet concentrations of oxazepam. Figure S3: Seasonal concentrations of 4 pharmaceuticals at Kristianstad WWTP. Figure S4: Estimated release of pharmaceuticals in kg. Figure S5: River sampling points. Figure S6: Lake sampling points. Figure S7: WWTPs by the coast. Table S1.a.: Information about the 8 WWTPs. Table S1.b. Complementary information about the eight WWTPs. Table S2: WWTP outlet concentrations. Table S3: Seasonal concentrations at Kristianstad WWTP. Table S4: Outlet masses from WWTPs. Table S5: Outlet wastewater volumes. Table S6: Upstream and downstream concentrations.

## Sample Availability

The physical wastewater samples and surface water sample are not available, as they were used during the sample preparation.

## References

[1] Halling-Sørensen B., Nors Nielsen S., Lanzky F., Ingerslev F., Holten Lützhøft S., Jørgensen S. (1998). Occurrence, fate and effects of pharmaceutical substances in the environment—A review. Chemosphere.

[2] Cleuvers M. (2004). Mixture toxicity of the anti-inflammatory drugs diclofenac, ibuprofen, naproxen, and acetylsalicylic acid. Ecotoxicol. Environ. Saf..

[3] Zhang Y., Geißen S.-U., Gal C. (2008). Carbamazepine and diclofenac: Removal in wastewater treatment plants and occurrence in water bodies. Chemosphere.

[4] Runnqvist H., Bak S., Hansen M., Styrishave B., Halling-Sørensen B., Björklund E. (2010). Determination of pharmaceuticals in environmental and biological matrices using pressurised liquid extraction—Are we developing sound extraction methods?. J. Chromatogr. A.

[5] Santos L., Araújo A., Fachini A., Pena A., Delerue-Matos C., Montenegro M. (2010). Ecotoxicological aspects related to the presence of pharmaceuticals in the aquatic environment. J. Hazard. Mater..

[6] Verlicchi P., Galletti A., Petrovic M., Barceló D. (2010). Hospital effluents as a source of emerging pollutants: An overview of micropollutants and sustainable treatment options. J. Hydrol..

[7] Vulliet E., Cren-Olivé C. (2011). Screening of pharmaceuticals and hormones at the regional scale, in surface and groundwaters intended to human consumption. Environ. Pollut..

[8] Martín J., Camacho-Munoz D., Santos J., Aparicio I., Alonso E. (2012). Occurrence of pharmaceutical compounds in wastewater and sludge from wastewater treatment plants: Removal and ecotoxicological impact of wastewater discharges and sludge disposal. J. Hazard. Mater..

[9] Arnold K., Boxall A., Ross Brown A., Cuthbert R., Gaw S., Hutchinson T., Jobling S., Madden J., Metcalfe C., Naidoo V. (2013). Assessing the exposure risk and impacts of pharmaceuticals in the environment on individuals and ecosystems. Biol. Lett..

[10] Li W. (2014). Occurrence, sources, and fate of pharmaceuticals in aquatic environment and soil. Environ. Pollut..

[11] Beek T., Weber F.-A., Bergmann A., Hickmann S., Ebert I., Hein A., Küster A. (2016). Pharmaceuticals in the environment—Global occurrence and perspectives. Environ. Toxicol. Chem..

[12] Daughton C. (2016). Pharmaceuticals in the environment (PiE): Evolution and impact of the published literature revealed by bibliometric analysis. Sci. Total Environ..

[13] Tlili I., Caria G., Ouddane B., Ghorbel-Abid I., Ternane R., Trabelsi-Ayadi M., Net S. (2016). Simultaneous detection of antibiotics and other drug residues in the dissolved and particulate phases of water by an off-line SPE combined with on-line SPE-LC-MS/MS: Method development and application. Sci. Total Environ..

[14] Wang J., Wang S. (2016). Removal of pharmaceuticals and personal care products (PPCPs) from wastewater: A review. J. Environ. Manag..

[15] Xie Z., Lu G., Li S., Nie Y., Ma B., Liu J. (2018). Behavioral and biochemical responses in freshwater fish Carassius auratus exposed to sertraline. Chemosphere.

[16] Brodin T., Fick J., Jonsson M., Klaminder J. (2013). Dilute Concentrations of a Psychiatric Drug Alter Behavior of Fish from Natural Populations. Science.

[17] Whitlock S., Glória Pereira M., Shore R., Lane J., Arnold K. (2018). Environmentally relevant exposure to an antidepressant alters courtship behaviours in a songbird. Chemosphere.

[18] Saaristo M., Brodin T., Balshine S., Bertram M., Brooks B., Ehlman S., McCallum E., Sih A., Sundin J., Wong B. (2018). Direct and indirect effects of chemical contaminants on the behaviour, ecology and evolution of wildlife. Proc. R. Soc. B.

[19] Escher B., Stapleton H., Schymanski E. (2020). Tracking complex mixtures of chemicals in our changing environment. Science.

[20] Pedrero F., Kalavrouziotis I., Alarcóna J., Koukoulakis P., Asanoc T. (2010). Use of treated municipal wastewater in irrigated agriculture—Review of some practices in Spain and Greece. Agric. Water Manag..

[21] Christou A., Agüera A., Maria Bayona J., Cytryn E., Fotopoulos V., Lambropoulou D., Manaia C., Michael C., Revitt M., Schröder P. (2017). The potential implications of reclaimed wastewater reuse for irrigation on the agricultural environment: The knowns and unknowns of the fate of antibiotics and antibiotic resistant bacteria and resistance genes—A review. Water Res..

[22] Moghaddam V., Changani F., Mohammadi A., Hadei M., Ashabi R., Ebrahimi Majd L., Hossein Mahvi A. (2017). Sustainable development of water resources based on wastewater reuse and upgrading of treatment plants: A review in the Middle East. Desalination Water Treat..

[23] SMHI—Sveriges Meteorologiska och Hydrologiska Institut. https://www.smhi.se/klimat/klimatet-da-och-nu/arets-vader/sommaren-2018-extremt-varm-och-solig-1.138134.

[24] Wahlberg C., Björlenius B., Paxéus N. (2010). Läkemedelsrester I Stockholms vattenmiljö—Förekomst, förebyggande åtgärder och rening av avloppsvatten. Stockholm Vatten.

[25] (2016). MistraPharma—Identification and Reduction of Environmental Risks Caused by Human Pharmaceuticals, MistraPharma Research 2008–2015. Final. Rep..

[26] Ågerstrand M., Berg C., Björlenius B., Breitholtz M., Brunström B., Fick J., Gunnarsson L., Larsson J., Sumpter J., Tysklind M. (2015). Improving Environmental Risk Assessment of Human Pharmaceuticals. Environ. Sci. Technol..

[27] Cimbritz M., Mattsson A. (2018). Reningstekniker för läkemedel och mikroföroreningar i avloppsvatten. Havs-Och Vattenmyndighetens Rapp..

[28] Sundin A.-M., Linderholm L., Hedlund B., Bly Joyce K., Klingspor K. (2017). Avancerad rening av avloppsvatten för avskiljning av läkemedelsrester och andra oönskade ämnen-Behov, teknik och konsekvenser. Nat. Rep..

[29] Mattson B., Andersson A., Ovesjö M.-L. (2015). Miljöindikatorer inom ramen för nationella läkemedelsstrategin (NLS). Rapport Frän CBL-Kansliet, Läkemedelsverket.

[30] Svahn O. (2016). Applied Environmental Analytical Chemistry for Monitoring and Measures against Antibiotics and Drug Residues in Vattenriket. Ph.D. Thesis.

[31] Svahn O., Björklund E. (2016). Increased electrospray ionization intensities and expanded chromatographic possibilities for emerging contaminants using mobile phases of different pH. J. Chromatogr. B.

[32] (2014). Läkemedelsrester i Avloppsvatten. Länsstyrelsen Skåne. TVL-Info.

[33] Wick A., Fink G., Joss A., Siegrist H., Ternes T. (2009). Fate of beta blockers and psycho-active drugs in conventional wastewater treatment. Water Res..

[34] Fick J., Lindberg R., Kaj L., Brorström-Lundén E. (2011). Results from the Swedish National Screening Programme 2010. Pharmaceuticals.

[35] Golovko O., Rehrl A.-L., Köhler S., Ahrens L. (2020). Organic micropollutants in water and sediment from Lake Mälaren, Sweden. Chemosphere.

[36] Zou H., Radke M., Kierkegaard A., McLachlan M. (2015). Temporal Variation of Chemical Persistence in a Swedish Lake Assessed by Benchmarking. Environ. Sci. Technol..

[37] Näslund J., Fick J., Asker N., Ekman E., Larsson J., Norrgren L. (2017). Diclofenac affects kidney histology in the three-spined stickleback (*Gasterosteus aculeatus*) at low μg/L concentrations. Aquat. Toxicol..

[38] Ringbom T., Salin K., Scholz B., Hillver S.-E., Ljung R. (2017). Tonvis med diklofenak i våra vatten–regeländring behövs. Läkartidningen.

[39] Loos R., Carvalho R., António D., Comero S., Locoro G., Tavazzi S., Paracchini B., Ghiani M., Lettieri T., Blaha L. (2013). EU-wide monitoring survey on emerging polar organic contaminants in wastewater treatment plant effluents. Water Res..

[40] Durán-Alvarez J., Prado B., González D., Sánchez Y., Jiménez-Cisneros B. (2015). Environmental fate of naproxen, carbamazepine and triclosan in wastewater, surface water and wastewater irrigated soil—Results of laboratory scale experiments. Sci. Total Environ..

[41] Björlenius B., Ripszámb M., Haglund P., Lindberg R., Tysklind M., Fick J. (2018). Pharmaceutical residues are widespread in Baltic Sea coastal and offshore waters–Screening for pharmaceuticals and modelling of environmental concentrations of carbamazepine. Sci. Total Environ..

[42] Daneshvar A., Aboulfadl K., Viglino L., Broséus R., Sauvé S., Madoux-Humery A.-S., Weyhenmeyer G., Prévost M. (2012). Evaluating pharmaceuticals and caffeine as indicators of fecal contamination in drinking water sources of the Greater Montreal region. Chemosphere.

[43] Sanzi Cortez F., da Silva Souza L., Lopes Guimarãe L., Emanoel Almeida J., Hermes Pusceddu F., Alves Maranho L., Gonçalves Mota L., Rodrigues Nobre C., Barbosa Moreno B., Moledo de Souza Abessa D. (2018). Ecotoxicological effects of losartan on the brown mussel *Perna perna* and its occurrence in seawater from Santos Bay (Brazil). Sci. Total Environ..

[44] Näslund J., Asker N., Fick J., Larsson J., Norrgren L. (2020). Naproxen affects multiple organs in fish but is still an environmentally better alternative to diclofenac. Aquat. Toxicol..

[45] Brodin T., Nordling J., Lagesson A., Klaminder J., Hellström G., Christensen B., Fick J. (2017). Environmental relevant levels of a benzodiazepine (oxazepam) alters important behavioral traits in a common planktivorous fish, (*Rutilus rutilus*). J. Toxicol. Environ. Health Part A.

[46] Fick J., Brodin T., Heyne M., Klaminder J., Jonsson M., Grabicova K., Randa T., Grabic R., Kodes V., Slobodnik J. (2017). Screening of benzodiazepines in thirty European rivers. Chemosphere.

[47] Östman M., Lindberg R., Fick J., Björn E., Tysklind M. (2017). Screening of biocides, metals and antibiotics in Swedish sewage sludge and wastewater. Water Res..

[48] Svahn O., Björklund E. (2019). Extraction Efficiency of a Commercial Espresso Machine Compared to a Stainless-Steel Column Pressurized Hot Water Extraction (PHWE) System for the Determination of 23 Pharmaceuticals, Antibiotics and Hormones in Sewage Sludge. Appl. Sci..

[49] Lindberg R., Wennberg P., Johansson M., Tysklind M., Andersson B. (2005). Screening of Human Antibiotic Substances and Determination of Weekly Mass Flows in Five Sewage Treatment Plants in Sweden. Environ. Sci. Technol..

[50] Thiebault T. (2020). Sulfamethoxazole/Trimethoprim ratio as a new marker in raw wastewaters: A critical review. Sci. Total Environ..

[51] Drzymała J., Kalka J. (2020). Ecotoxic interactions between pharmaceuticals in mixtures: Diclofenac and sulfamethoxazole. Chemosphere.

[52] Östman M., Fick J., Näsström E., Lindberg H. (2014). A snapshot of illicit drug use in Sweden acquired through sewage water analysis. Sci. Total Environ..

[53] Svahn O., Björklund E. (2017). Interkalibrerad Läkemedelsanalys 2017—Ett Samarbetsprojekt för Ökad Analyskvalité.

[54] Kellner M., Porseryd T., Porsch-Hällström I., Hansen S., Olsén K. (2015). Environmentally relevant concentrations of citalopram partially inhibit feeding in the three-spine stickleback (*Gasterosteus aculeatus*). Aquat. Toxicol..

[55] Kellner M., Porseryd T., Porsch-Hällström I., Borg B., Hansen S., Roufidou C., Olsén K. (2018). Developmental exposure to the SSRI citalopram causes long-lasting behavioural effects in the three-spined stickleback (*Gasterosteus aculeatus*). Ecotoxicology.

[56] Lindberg R., Fick J., Tysklind M. (2010). Screening of antimycotics in Swedish sewage treatment plants—Waters and sludge. Water Res..

[57] Hedgespeth M., Karasek T., Ahlgren J., Berglund O., Brönmark C. (2018). Behaviour of freshwater snails (*Radix balthica*) exposed to the pharmaceutical sertraline under simulated predation risk. Ecotoxicology.

[58] Hedgespeth M., Nilsson A., Berglund O. (2014). Ecological implications of altered fish foraging after exposure to an antidepressant pharmaceutical. Aquat. Toxicol..

[59] Parkkonen J., Larsson J., Adolfsson-Erici M., Pettersson M., Berg A., Olsson P., Förlin L. (2000). Contraceptive pill residues in sewage effluent are estrogenic to fish. Mar. Environ. Res..

[60] Svensson J., Fick J., Brandt I., Brunström B. (2014). Environmental concentrations of an androgenic progestin disrupts the seasonal breeding cycle in male three-spined stickleback (*Gasterosteus aculeatus*). Aquat. Toxicol..

[61] Statistics Sweden. https://www.scb.se/hitta-statistik/statistik-efter-amne/befolkning/befolkningens-sammansattning/befolkningsstatistik/pong/tabell-och-diagram/helarsstatistik--forsamling-landskap-och-stad/folkmangd-i-landskapen-den-31-december-2016/.

[62] Olshammar M., Ek M., Rosenquist L., Ejhed H., Sidvall A., Svanström S. (2015). Uppdatering Av Kunskapsläget Och Statistik för Små Avloppsanläggningar.

[63] Kommittén för Samordnad Kontroll av Helgeån (2012). Med Långtidsdiagram 1973–2011.

[64] (2005). Klippans Läderfabrik. Kompletterande undersökningar av Bäljane Å 2005. Rapport Klippans Kommun.

[65] (1995). Miljöövervakning Länsstyrelsen i Kristianstads Län. Klammersbäck, Mölleån, Rörums norra å, Rörums södra å, Kvarnbybäcken. Österlen-Åar–Underl..

[66] http://www.segea.se/Om-Segea.html.

[67] https://vattenriket.kristianstad.se/other-languages/english/.

[68] Björklund E., Svahn O., Bak S., Oppong Bekoe S., Hansen M. (2016). Pharmaceutical Residues Affecting the UNESCO Biosphere Reserve Kristianstads Vattenrike Wetlands: Sources and Sinks. Arch. Environ. Contam. Toxicol..

[69] U.S. Environmental Protection Agency, Office of Water, Office of Science and Technology Engineering and Analysis Division (2007). US EPA Method 1694: Pharmaceuticals and Personal Care Products in Water, Soil, Sediment, and Biosolids by HPLC/MS/MS.

[70] https://vattenriket.kristianstad.se/vramsan-vattendrag/.

[71] Dunca E., Söderberg H., Norrgrann O. (2011). Shell growth and age determination in the freshwater pearl mussel Margaritifera margaritifera in Sweden: Natural versus limed streams. Ferrantia.

[72] Hartmut F., Gerstmann S. (2007). Declining Populations of Freshwater Pearl Mussels (Margaritifera margaritifera) Are Burdened with Heavy Metals and DDT/DDE. AMBIO: A J. Hum. Environ..

