# Supplementary material for: Total Release of 21 Indicator Pharmaceuticals Listed by the Swedish Medical Products Agency from Wastewater Treatment Plants to Surface Water Bodies in the 1.3 Million Populated County Skåne (Scania), Sweden
Source: Molecules. 2021 Dec 23;27(1):77. doi: 10.3390/molecules27010077 (PMC8746806; doi:10.3390/molecules27010077)
Supplement: Supplementary file 1 [file molecules-27-00077-s001.zip › molecules-1491196-supplementary-final (1).pdf]

molecules

MDPI

Supplementary Materials

Total Release of 21 Indicator Pharmaceuticals Listed by the Swedish Medical Products Agency from Wastewater Treatment Plants to Surface Water Bodies in the 1.3 Million Populated County Skåne (Scania), Sweden

Erland Björklund * and Ola Svahn *

Department of Environmental Science and Bioscience, Kristianstad University, Elmetorpsvägen 15, SE-291 88, Kristianstad, Sweden
* Correspondence: erland.bjorklund@hkr.se (E.B.); ola.svahn@hkr.se (O.S.)

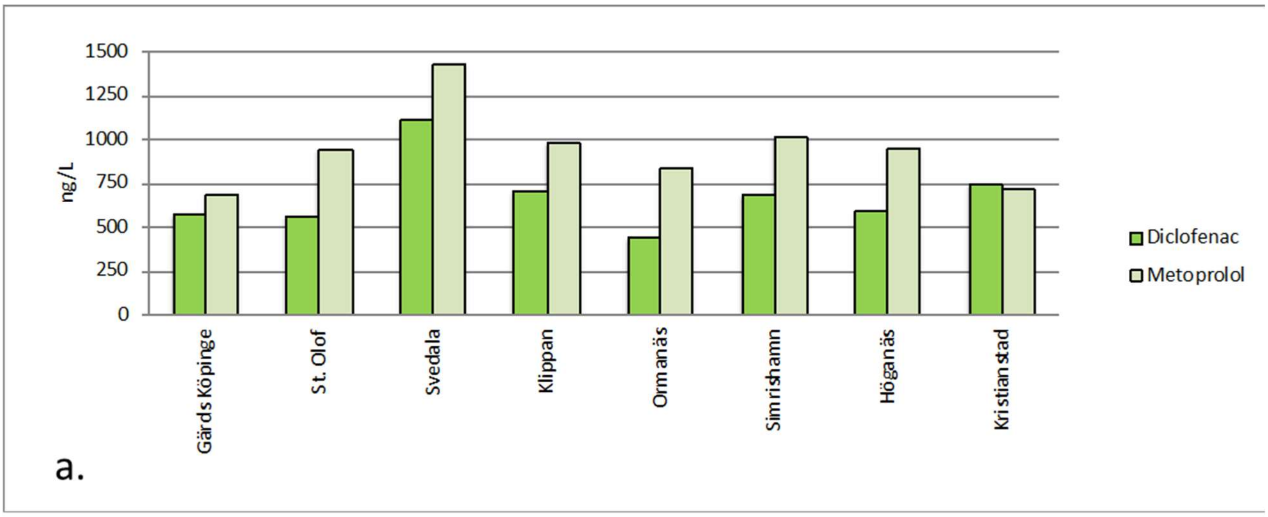

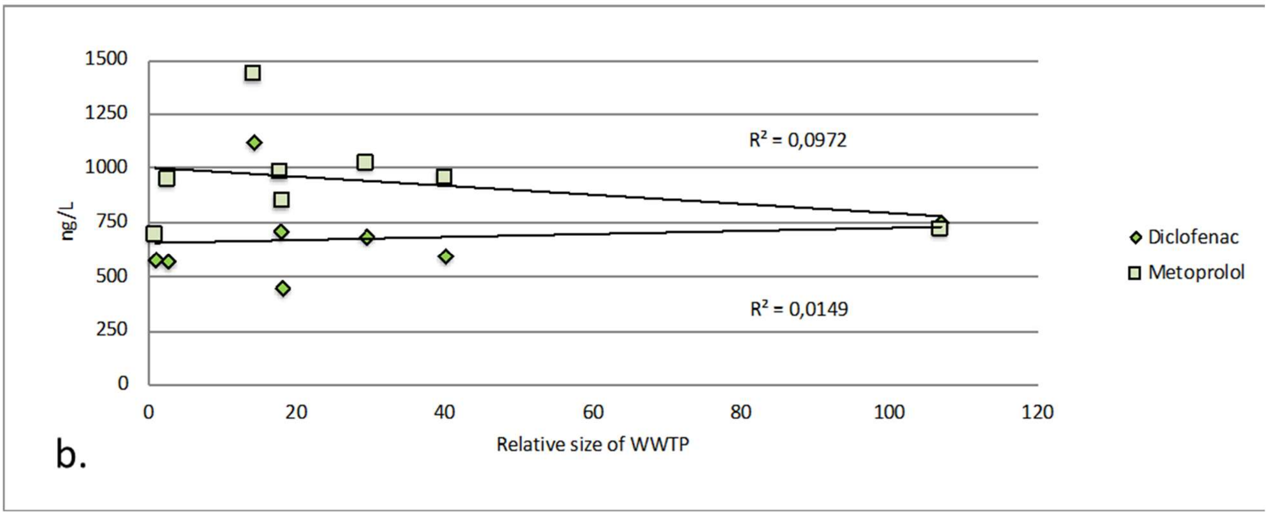

Figure S1. Outlet concentrations of diclofenac and metoprolol.

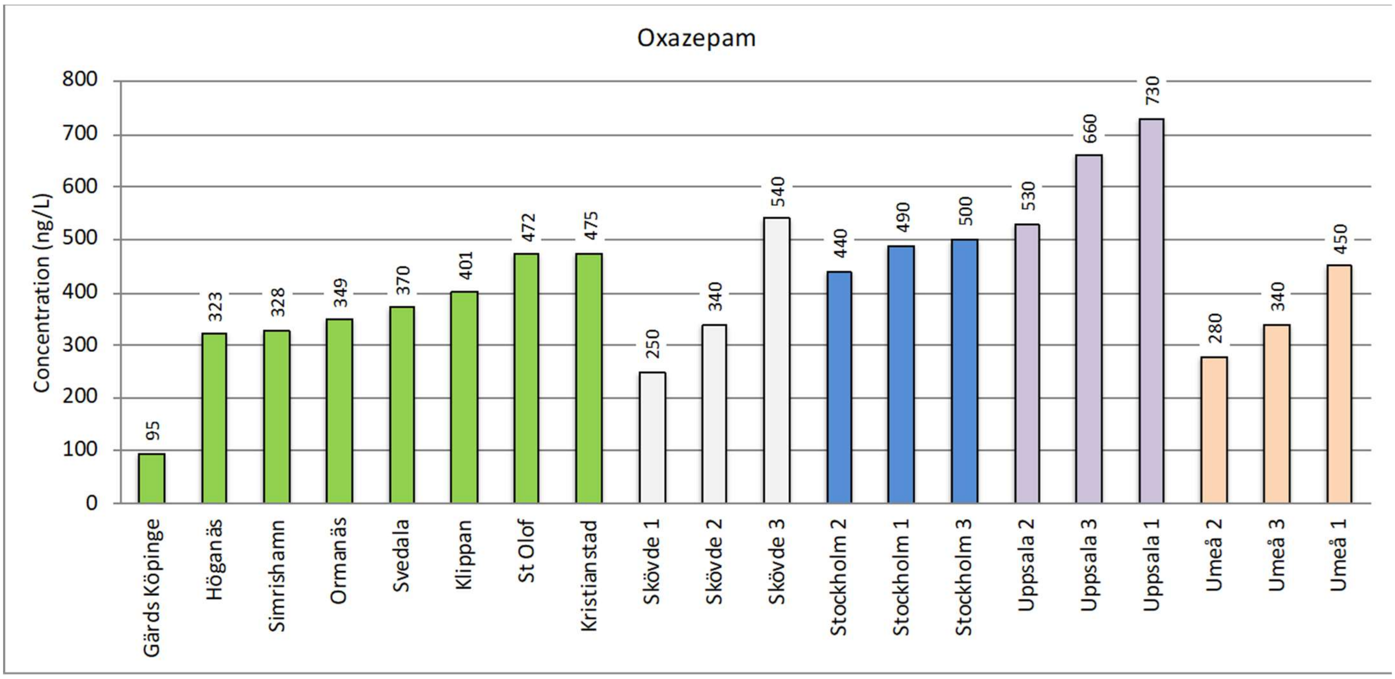

Figure S2. Outlet concentrations of oxazepam.

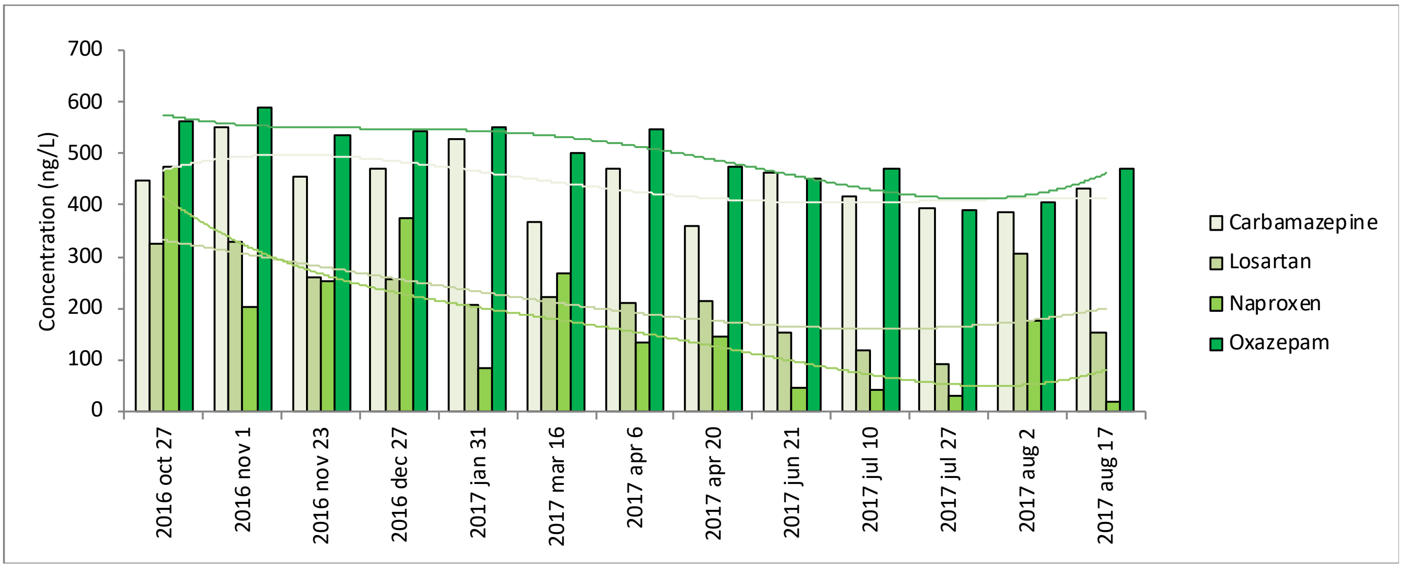

Figure S3. Seasonal concentrations of 4 pharmaceuticals at Kristianstad WWTP.

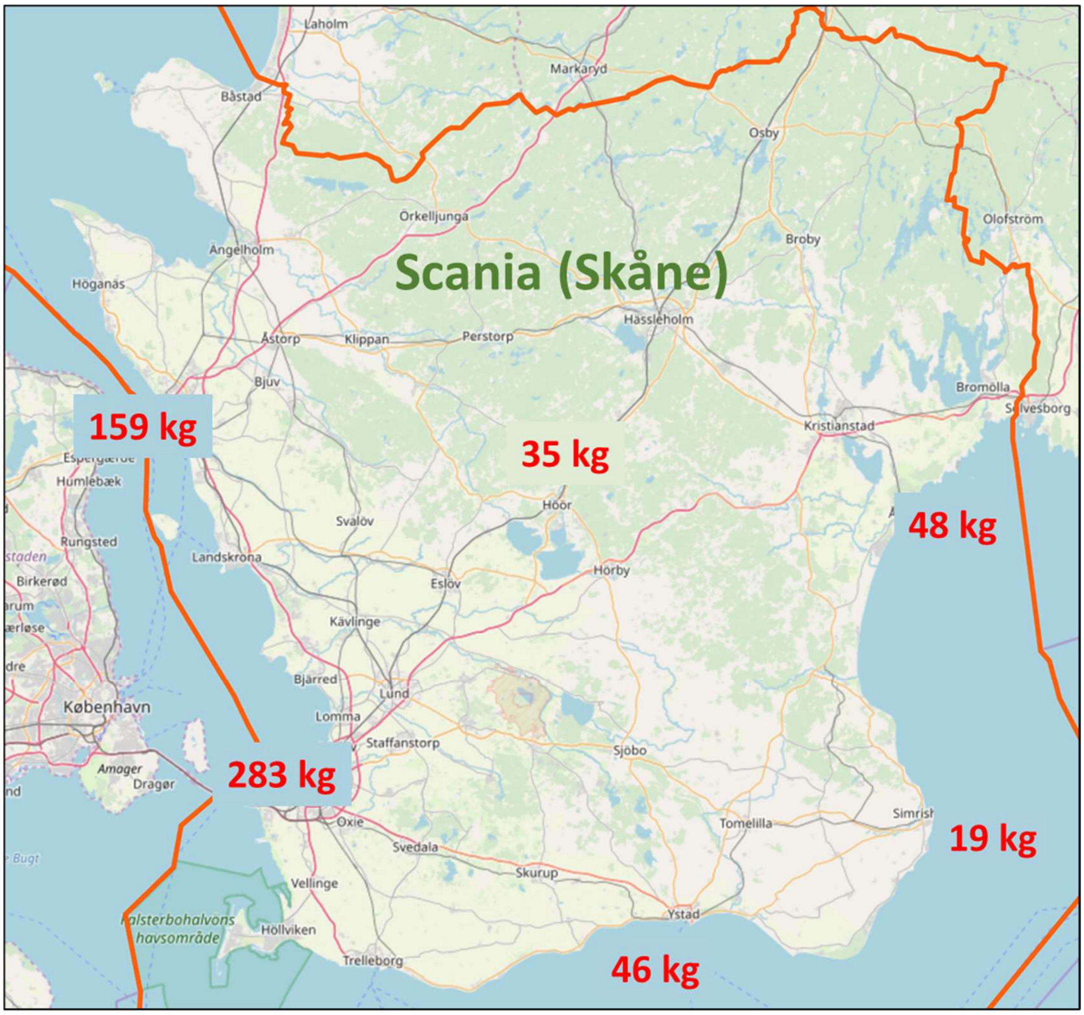

Figure S4. Estimated release of pharmaceuticals in kg.

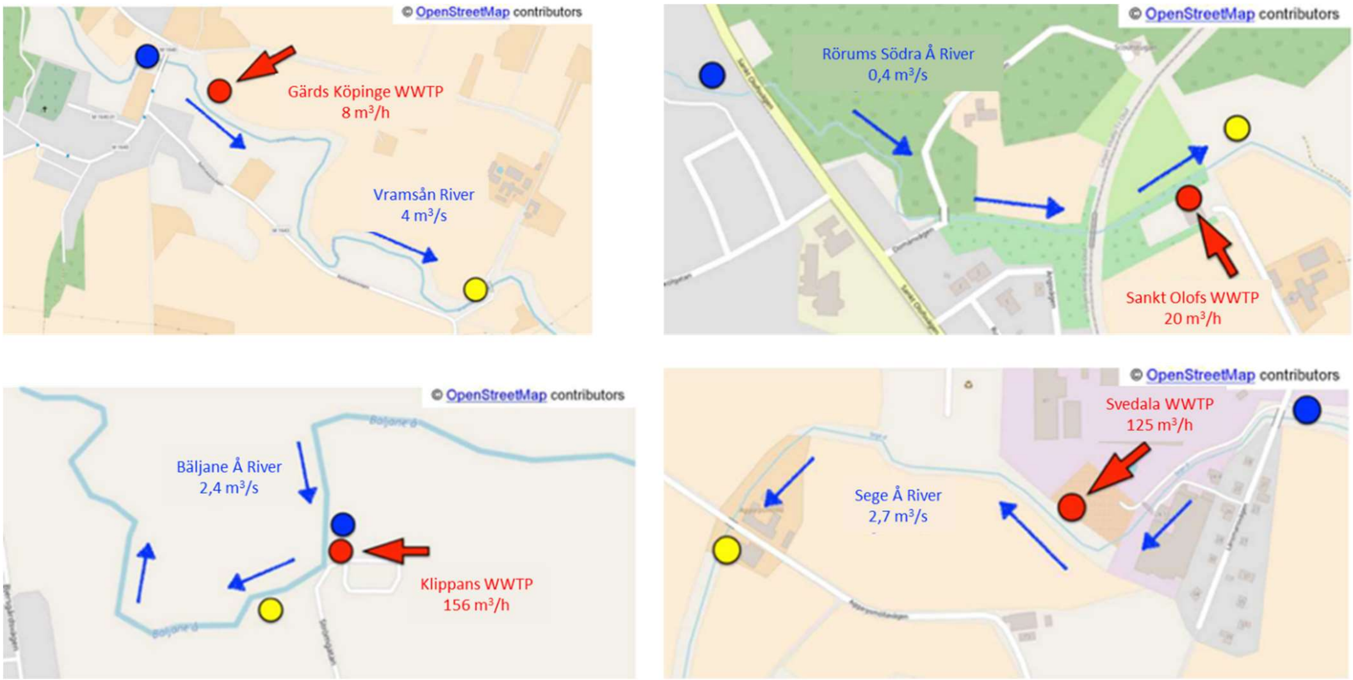

Figure S5. River sampling points.

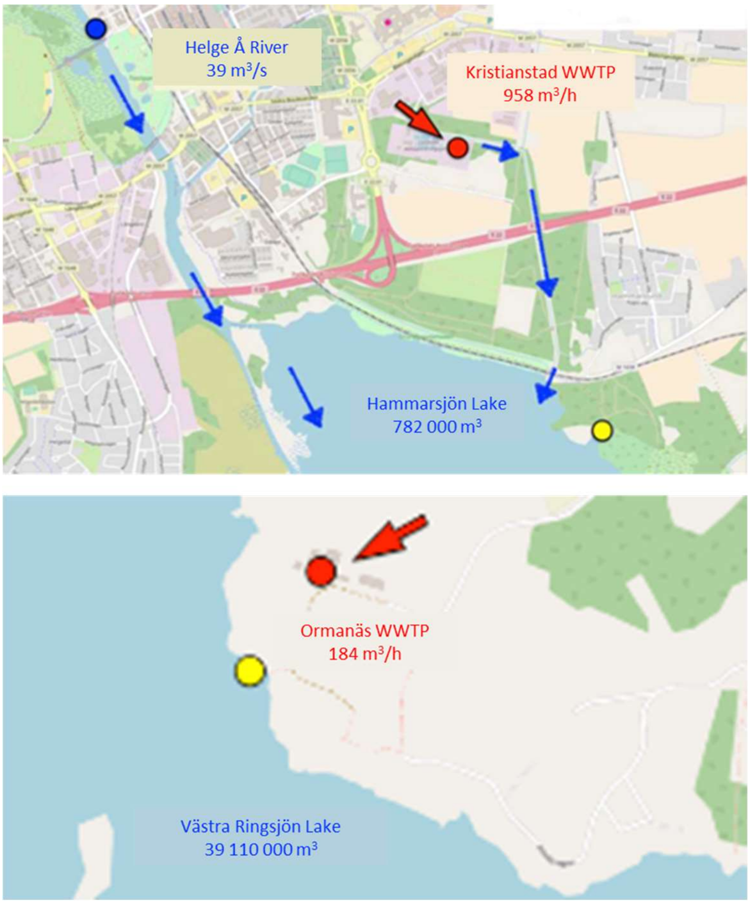

Figure S6. Lake sampling points.

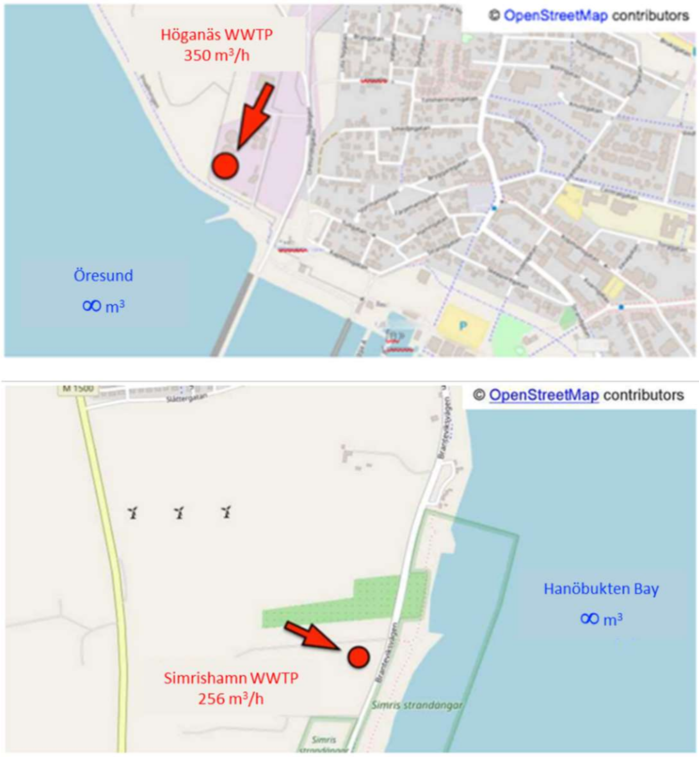

Figure S7. WWTPs by the coast.

Table S1.a. Information about the 8 WWTPs.

WWTP	Annual Volume m³	Year	Average Hourly Flow m³/h	Max. Dimension PE	Actual Number PE	Residents Connected PE	Industry Connected PE	Other Activities PE	Recipient
**Gärds Köpinge**	76 538	2016	8.75	900	425	?	0	0	Vramsån river
**Höganäs**	3 075 792	2016	350	35 000	20 257	23 033	200	0	Öresund sea
**Klippan**	1 366 560	2016	156	17 000	8 600	13 000	0	0	Bäljane Å river
**Kristianstad**	8 186 000	2016	958	205 000	118 300	52 000	64 000	?	Hammarsjön lake
**Ormanäs**	1 388 399	2016	184	13 500	3 782	9 000	?	2 681	Västra Ringsjön lake
**Simrishamn**	2 250 000	?	256	?	87 000	23 000	6 740	?	Hanöbukten Baltic sea
**Sankt Olof**	200 000	?	?	1 000	600	?	?	?	Rörums Södra Å river
**Svedala**	1 100 164	2016	125	18 500	9 800	12 000	?	Sturup air-port	Sege Å river

Operating parameters according to the responses returned by the participating organisations in the completed questionnaires. The value of zero ("0") means that the organisations stated this value, while the question marks ("?") mean that no value was given or that uncertainty exists.

Table S1.b. Complementary information about the eight WWTPs.

WWTP	Step 1	Step 2	Step 3	Step 4	Step 5	Step 6	Step 7
**Simrishamn**	Mechanical, Cleaning grate, sand trap	Chemical precipitation	Biological Anox/contact tanks	Reed beds	Final sedimentation		
**Svedala**	Inlet pumps/ grating/ sand trap	2 x biolines with pre-denitrification	Intermediate sedimentation	Ferric chloride dosing	Final sedimentation	Sludge layer, gravitation thickener, centrifuge, liming of sludge (reserve sludge treatment is reed beds)	
**Ormanäs**	Mono-screen grating	Preliminary sedimentation	Activated sludge/ Pre-denitrification /Anox	Intermediate sedimentation	Chemical stage/precipitation PAX-XL 100	Floration tanks	Sand filter
**Klippan**	Stair grating	Preliminary sedimentation	Activated sludge/ Pre-denitrification	Simultaneous precipitation with iron sulphate		Sedimentation	
**Höganäs**	Step screen	Preliminary sedimentation	Activated sludge process/ Pre-denitrification/ bio-p	Post precipitation with polyaluminium chloride, sedimentation	Downstream sand filter		
**Gärds Köpinge**	Mechanical cleaning: Spiral screen	Activated sludge/ Aeration (pipe aerator)/ Sedimentation	Chemical precipitation with $FeCl_3$/Flocking	Sedimentation			
**Kristianstad**	Stair grating	Sedimentation	Activated sludge/ Pre-denitrification/ Krauss process/ Sedimentation	Activated sludge/ Pre-denitrification/ Sedimentation	Precipitation with $FeCI_3$/ Flocking	Slat sedimentation	Downstream sand filter

Operating parameters according to the responses returned by the participating organisations in the completed questionnaires.

Table S2. WWTP outlet concentrations.

Pharmaceutical	LOQ (ng/L)	Gärds Köpinge WWTP	Höganäs WWTP	Klippan WWTP	Kristianstad WWTP	Ormanäs WWTP	Simrishamn WWTP	Sankt Olof WWTP	Svedala WWTP	Average Concentration	Standard Deviation	*RSD (%)*
Ciprofloxacin	10	<LOQ	<LOQ	<LOQ	<LOQ	<LOQ	<LOQ	<LOQ	<LOQ	<LOQ		
Citalopram	1	120	93	135	80	164	110	104	217	**128**	44	*35*
Clarithromycin	2	<LOQ	56	101	24	82	29	3	213	**64**	70	*111*
Diclofenac	2	579	594	707	746	442	685	569	1117	**680**	201	*30*
Erythromycin	0.5	1	53	166	215	12	113	3	640	**150**	213	*142*
Estrone	0.2	18	1	1	3	63	7	4	7	**13**	21	*161*
Fluconazole	0.3	3	59	52	105	71	24	17	53	**48**	33	*68*
Ibuprofen	100	<LOQ	<LOQ	92	<LOQ	1158	613	124	107	**262**	415	*158*
Carbamazepine	0.5	139	442	439	470	529	233	601	699	**444**	183	*41*
Ketoconazole	15	<LOQ	<LOQ	<LOQ	<LOQ	<LOQ	<LOQ	<LOQ	<LOQ	<LOQ		
Levonorgestrel	10	<LOQ	<LOQ	<LOQ	<LOQ	<LOQ	<LOQ	<LOQ	<LOQ	<LOQ		
Losartan	1	386	344	274	217	83	673	497	921	**424**	268	*63*
Metoprolol	2	692	954	979	714	843	1016	943	1430	**946**	230	*24*
Methotrexate	2	<LOQ	<LOQ	<LOQ	<LOQ	<LOQ	<LOQ	<LOQ	<LOQ	<LOQ		
Naproxen	10	145	378	290	119	266	379	1430	304	**414**	421	*102*
Oxazepam	1	95	323	401	475	349	328	472	370	**352**	119	*34*
Sertraline	1	19	18	40	4	47	8	27	32	**24**	15	*62*

Sulfamethoxazole	2	<LOQ	238	118	208	173	51	<LOQ	281	**134**	109	*81*
Tramadol	10	190	145	187	208	118	81	94	151	**147**	47	*32*
Trimethoprim	1	6	95	78	29	64	33	1	107	**52**	40	*78*
Zolpidem	0.5	3	3	2	3	1	1	2	4	**2**	1	*45*

Outlet concentrations in **ng/L** of 21 pharmaceuticals from eight Scanian wastewater treatment plants (WWTPs). **BLUE** values indicate the treatment plant with the lowest outlet concentration, while **RED** values indicate the treatment plant with the highest outlet concentration of the respective substance. **ORANGE** cells indicate antibiotics.

Table S3. Seasonal concentrations at Kristianstad WWTP.

**Pharmaceutical**	**2016 Oct 27**	**2016 Nov 1**	**2016 Nov 23**	**2016 Dec 27**	**2017 Jan 31**	**2017 Mar 16**	**2017 Apr 6**	**2017 Apr 20**	**2017 Jun 21**	**2017 Jul 10**	**2017 Jul 27**	**2017 Aug 2**	**2017 Aug 17**	**Average Concentration**	**Standard Deviation**	***RSD (%)***
Metoprolol	774	724	659	855	892	684	691	616	481	421	541	478	481	**638**	145	*23*
Diclofenac	658	781	781	836	898	770	789	622	515	407	359	614	485	**655**	165	*25*
Carbamazepine	447	552	454	470	529	367	472	360	461	417	395	385	431	**441**	56	*13*
Losartan	326	330	261	256	207	221	212	213	153	119	91	306	153	**219**	73	*33*
Naproxen	476	204	253	376	85	269	132	144	44	41	32	177	20	**173**	135	*78*
Oxazepam	561	589	535	544	551	501	549	474	451	471	389	406	472	**499**	59	*12*

Measured concentrations in ng/L of 6 pharmaceuticals in outlet water from Kristianstad WWTP at different seasons in 2016-2017.

Table S4. Outlet masses from WWTPs.

Pharmaceutical	Gärds Kö-pinge	Sankt Olof	Svedala	Klippan	Or-manäs	Simri-shamn	Höganäs	Kristian-stad	Total (g/year)
Ciprofloxacin	0	0	0	0	0	0	0	0	0
Citalopram	9	21	239	185	227	247	286	659	1873
Clarithromycin	0	1	235	139	113	64	172	196	920
Diclofenac	44	114	1228	966	613	1541	1827	6107	12440
Erythromycin	0	1	704	227	17	254	162	1761	3127
Estrone	1	1	7	1	88	16	4	20	138
Fluconazole	0	3	59	71	98	55	182	860	1328
Ibuprofen	0	25	117	125	1608	1380	0	0	3255
Carbamazepine	11	120	769	600	735	525	1361	3844	7964
Ketoconazole	0	0	0	0	0	0	0	0	0
Levonorgestrel	0	0	0	0	0	0	0	0	0
Losartan	30	99	1013	374	116	1513	1058	1772	5975
Metoprolol	53	189	1573	1338	1171	2286	2935	5847	15392
Methotrexate	0	0	0	0	0	0	0	0	0
Naproxen	11	286	334	397	370	852	1162	976	4389
Oxazepam	7	94	407	548	484	737	993	3888	7159
Sertraline	1	5	36	54	66	19	55	31	267
Sulfamethoxa-zole	0	0	309	162	240	115	732	1704	3261
Tramadol	15	19	167	256	163	183	444	1704	2950
Trimethoprim	0	0	118	106	89	74	293	241	921
Zolpidem	0	0	5	3	1	3	9	29	50
Total (g)	184	980	7328	5558	6206	9868	11681	29638	71442
Total (kg)	0.2	1.0	7.3	5.6	6.2	9.9	11.7	29.6	71.4

Outlet masses in grams of 21 pharmaceuticals from eight Scanian wastewater treatment plants (listed in size). **ORANGE** cells indicate antibiotics.

Table S5. Outlet wastewater volumes.

NSVA		
**WWTP**	**Volume m³/year**	**Mass kg/year**
Röstånga	10 8040	0.41
Kvidinge	12 0815	0.45
Kågeröd	33 6895	1.27
Eketorp	495 305	1.86
Svalöv	535 455	2.01
Ekebro	1 111 790	4.18
Torekovs	1 182 235	4.45
Nyvångsverket	1 334 805	5.02
Lundåkraverket	4 581 115	17.22
Öresundsverket	23 904 215	89.88
**Total**	**33 710 670**	**126.75**
**VA SYD**		
**WWTP**	**Volume m³/year**	**Mass kg/year**
Örtofta	33 800	0.13
Torna Hällestad	46 500	0.17
Stockamöllan	65 100	0.24
Hurva	88 430	0.33
Håstad	98 373	0.37
Revinge	106 158	0.40
Billinge	141 800	0.53
Löberöd	152 200	0.57
Flyinge	206 000	0.77
Stehag	299 756	1.13
Veberöd	340 500	1.28
Södra Sandby	830 000	3.12
Ellinge	4 433 000	16.67
Klagshamn	8 305 000	31.23
Källby	11 290 000	42.45
Sjölundaverket	42 258 000	158.89
**Total**	**68 694 617**	**258.29**
**VA SYD**		
**WWTP**	**Volume m³/year**	**Mass kg/year**
Örtofta	33 800	0.13
Torna Hällestad	46 500	0.17
Stockamöllan	65 100	0.24
Hurva	88 430	0.33

Håstad	98 373	0.37
Revinge	106 158	0.40
Billinge	141 800	0.53
Löberöd	152 200	0.57
Flyinge	206 000	0.77
Stehag	299 756	1.13
Veberöd	340 500	1.28
Södra Sandby	830 000	3.12
Ellinge	4 433 000	16.67
Klagshamn	8 305 000	31.23
Källby	11 290 000	42.45
Sjölundaverket	42 258 000	158.89
**Total**	**68 694 617**	**258.29**

Outlet wastewater volumes and estimated masses in kilograms of 21 pharmaceuticals from various wastewater treatment organisations in Scania.

Table S6. Upstream and downstream concentrations.

Pharmaceutical	LOQ (ng/L)	Gärds Köpinge Vramsån River		Klippan WWTP Bäljane Å River		Sankt Olof WWTP Rörums Södra Å River		Svedala WWTP Sege Å River		Kristianstad WWTP Helge Å River / Hammarsjön Lake		Ormanäs WWTP Ringsjön Lake
		Upstream	Downstream	Upstream	Downstream	Upstream	Downstream	Upstream	Downstream	Upstream	Downstream	Downstream
Ciprofloxacin	10	<LOQ	<LOQ	<LOQ	<LOQ	<LOQ	<LOQ	<LOQ	<LOQ	<LOQ	<LOQ	<LOQ
Citalopram	1	<LOQ	<LOQ	<LOQ	2.8	<LOQ	2.0	<LOQ	6.2	<LOQ	5.1	<LOQ
Clarithromycin	2	<LOQ	<LOQ	<LOQ	2.0	<LOQ	<LOQ	<LOQ	7.1	<LOQ	2.7	<LOQ
Diclofenac	2	4.3	5.0	2.3	41	<LOQ	25	<LOQ	57	3.6	59	<LOQ
Erythromycin	0.5	0.6	0.8	1.4	8.8	<LOQ	<LOQ	<LOQ	33	0.8	24	0.5
Estrone	0.2	0.3	0.4	0.2	0.2	0.2	0.3	0.2	0.4	<LOQ	0.2	0.6
Fluconazole	0.3	0.3	<LOQ	0.6	2.0	<LOQ	0.4	<LOQ	1.4	0.6	6.1	1.2
Ibuprofen	100	<LOQ	<LOQ	<LOQ	<LOQ	<LOQ	<LOQ	<LOQ	<LOQ	<LOQ	<LOQ	<LOQ
Carbamazepine	0.5	1.5	1.8	6.7	27	<LOQ	25	<LOQ	38	5.9	59	12
Ketoconazole	15	<LOQ	<LOQ	<LOQ	<LOQ	<LOQ	<LOQ	<LOQ	<LOQ	<LOQ	<LOQ	<LOQ
Levonorgestrel	-	<LOQ	<LOQ	<LOQ	<LOQ	<LOQ	<LOQ	<LOQ	<LOQ	<LOQ	<LOQ	<LOQ
Losartan	1	1.2	1.7	1.9	12	<LOQ	19	<LOQ	33	2.2	16	1.7
Metoprolol	2	4.8	5.7	7.7	52	<LOQ	34	<LOQ	73	6.0	62	3.9

Methotrexate	2	*<LOQ*	*<LOQ*	*<LOQ*	*<LOQ*	*<LOQ*	*<LOQ*	*<LOQ*	*<LOQ*	*<LOQ*	*<LOQ*	*<LOQ*
Naproxen	10	*<LOQ*	*<LOQ*	*<LOQ*	13	*<LOQ*	57	3.2	12	*<LOQ*	*<LOQ*	*<LOQ*
Oxazepam	1	3.4	3.7	5.6	23	*<LOQ*	17	*<LOQ*	18	3.8	56	4.7
Sertraline	1	*<LOQ*	*<LOQ*	*<LOQ*	*<LOQ*	*<LOQ*	*<LOQ*	*<LOQ*	1.6	*<LOQ*	*<LOQ*	*<LOQ*
Sulfamethoxazole	2	*<LOQ*	*<LOQ*	4.0	11	*<LOQ*	*<LOQ*	*<LOQ*	11	1.9	34	3.4
Tramadol	10	*<LOQ*	*<LOQ*	*<LOQ*	11	*<LOQ*	*<LOQ*	*<LOQ*	8.8	*<LOQ*	26	*<LOQ*
Trimethoprim	1	*<LOQ*	*<LOQ*	*<LOQ*	3.3	1.7	1.3	*<LOQ*	5.4	0.9	2.3	*<LOQ*
Zolpidem	0.5	*<LOQ*	*<LOQ*	*<LOQ*	*<LOQ*	*<LOQ*	*<LOQ*	*<LOQ*	*<LOQ*	*<LOQ*	*<LOQ*	*<LOQ*

Concentrations upstream and downstream WWTPs in ng/L of 21 pharmaceuticals in Scanian recipients. **ORANGE** cells indicate antibiotics.
